# The Tra/Dsx-JHBP axis controls female-specific gene expression and oviposition in locusts

**DOI:** 10.1371/journal.pbio.3003321

**Published:** 2025-08-05

**Authors:** Xiaoming Zhao, Qian Ma, Ti Shao, Pan Jia, Zhaochen Wu, Caiyan Huo, Yuxin Wang, Weimin Liu, Zhangwu Zhao, Chen-Zhu Wang, Sheng Li, Jianzhen Zhang

**Affiliations:** 1 Shanxi Key Laboratory of Nucleic Acid Biopesticides, Institute of Applied Biology, Shanxi University, Taiyuan, Shanxi, China; 2 College of Life Science, Shanxi University, Taiyuan, Shanxi, China; 3 State Key Laboratory of Integrated Management of Pest Insects and Rodents, Institute of Zoology, Chinese Academy of Sciences, Beijing, China; 4 Guangdong Provincial Key Laboratory of Insect Developmental Biology and Applied Technology, Guangzhou Key Laboratory of Insect Development Regulation and Application Research, Institute of Insect Science and Technology & School of Life Sciences, South China Normal University, Guangzhou, China; Centre National de la Recherche Scientifique, FRANCE

## Abstract

Sexual dimorphism is a crucial aspect of morphological and behavioral traits in animals. Unlike males, adult female locusts, i.e., *Locusta migratoria*, have highly extensible abdominal intersegmental membranes (ISMs) that facilitate deep oviposition into the soil, displaying an iconic sexual dimorphism, but the underlying mechanisms remain largely unknown. Here, we reveal that the extremely extensible ISMs in adult females are predominantly controlled by two female-specific proteins, LmAbd-1 and LmAbd-6, ensuring the oviposition behavior. Moreover, we discovered that LmJHBP, a juvenile hormone (JH) binding protein specifically expressed in adult female ISMs, mediates JH signaling to induce *LmAbd-1* and *LmAbd-6* expression. Importantly, the sex differentiation pathway (i.e., *Tra-2* and *Dsx*) determines the female-specific expression pattern of *LmJHBP,* and thus those of JH signaling and *LmAbd-1* and *LmAbd-6* expression. The finding of Tra/Dsx-JHBP axis significantly advanced understanding of sexual dimorphism and the adaptation of oviposition behavior in insects, the evolutionarily successful “segmentation” animals.

## Abstract 2

Female locusts have highly extensible abdominal intersegmental membranes that facilitate deep oviposition into the soil, and represent an iconic sexual dimorphism. This study reveals that the Tra/Dsx-JHBP pathway regulates the expression pattern of two female-specific proteins that induce this oviposition behavior.

## Introduction

Sexual dimorphism is ubiquitous in the animal kingdom, leading to sex-specific phenotypic diversity, including those of morphological, behavioral, and life-history traits [1]. In insects, morphological diversity in specific organs between males and females of the same species are associated with body size, development time, feeding strategy, and other characteristics [2–5]. Male and female insects exhibit distinct morphological and physiological characteristics in their reproductive systems, which are intricately adapted to their respective reproductive roles. Among these, the female reproductive system is particularly specialized for egg production and oviposition behavior. This critical behavior requires the collaboration of different organs and are regulated by both environmental conditions and intrinsic signals [6,7].

“Parents’ love for their children is far-reaching”. As implied by a saying in the “Warring States” in ancient China, although most insect mothers do not “nurture” their offspring (they die quietly after egg laying), they still have a unique way of expressing “motherly love”. Female insects have evolved a variety of egg-laying strategies for facilitating the growth and development of offspring and population thriving; thus, their selection of oviposition sites is diverse, including soil, plant roots, stems, leaves, and fruits, decaying wood, insect larvae, and substrates such as water [8,9]. Locusts, a subset of grasshopper species, are notable for their swarming, long-distance migratory and extreme egg-laying behaviors, and locust plagues have been viewed as one of the most devastating natural disasters since the dawn of agrarian civilization [10,11]. Adult female locusts, with remarkable oviposition site preference, typically deposit their egg pods into moist and loose soil to prevent water loss and predation by predators. Notably, this process critically depends on the abdominal intersegmental membranes (ISMs), which allow adult females to extend their abdomen deep into the soil for egg-laying [12,13]. The abdominal ISM is located between two adjacent segments (Seg) on the abdomen of an insect, which exhibits extraordinary extensibility in adult female locusts. The development of this trait is closely tied to juvenile hormone (JH), as removal of the corpora allata, the glandular organ responsible for JH production, prevents the abdominal ISM extensibility [14]. Nevertheless, over the past few decades, the intricate mechanisms underlying how adult female locusts develop this egg-laying strategy have remained largely unexplored.

The cuticle, also known as the exoskeleton, is the first and major barrier, protecting the insect from penetration of external compounds [15]. The cuticle structure including segmental cuticle is generally well preserved among insect species and consists of different layers (epicuticle, hard exocuticle, untanned endocuticle, and epidermal cell layer). However, ISMs do not have a hard exocuticle between epicuticle and endocuticle, showing common features yet remarkable differences from the segmental cuticle [16,17]. Although adult locusts of both sexes have ISMs, there are significant structural differences between females and males [16], displaying an iconic sexual dimorphism. The adult female ISMs in locusts are markedly thicker and possess specialized features, including a lamella epicuticle and a highly folded structure. Additionally, the untanned endocuticle in adult female ISMs exhibits a spiral lamella and elastomer layer structure, likely contributing to their remarkable extensibility [18]. Like all the insect cuticle, ISMs are predominantly composed of chitin filaments and proteins, especially structural cuticle proteins (CPs) [14,19–21]. These substances are deposited in the specific layers to modulate the cuticle’s physical properties, which vary across developmental stages and in different regions due to chitin-protein interactions [22–24]. Interestingly, the orientation of chitin fibers in ISMs is perpendicular to their stretching direction suggesting that stiffness is not directly influenced by chitin. Instead, the extensibility of ISMs may be governed by modifications in the protein matrix [13,25,26]. However, the key structural CPs that control the formation of sexually dimorphic ISMs remain never discovered in insects.

The migratory locust, *Locusta migratoria*, a globally significant agricultural pest, has long served as a model organism for studies on insect morphology, behavior, and physiology [27,28]. In the present study, we identified two major structural CP genes in adult female locusts that are critical for ISMs extensibility and subsequently oviposition behavior. Furthermore, we demonstrated that their expression levels are induced by the sex differentiation pathway and JH signaling. The discoveries provide a significant advancement for understanding sexual dimorphism and adaptive behaviors in the evolutionarily successful “segmentation” insects.

## Results

### Abdominal ISMs display sexual dimorphism in adult locusts

**Fig 1:**
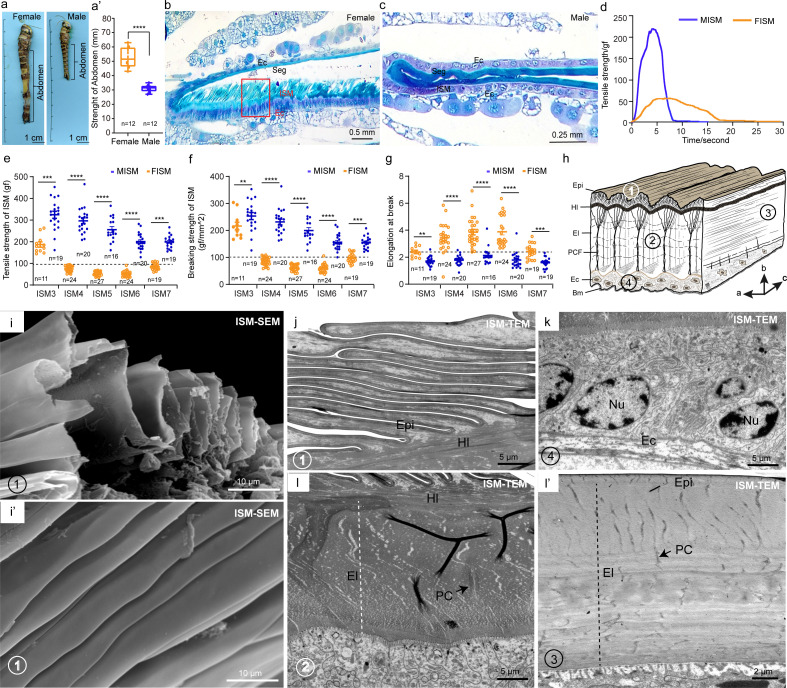
Dimorphism of the intersegmental membrane in adult female and male locusts. **(a, a′)** The stretching lengths of abdomen in adult female and male locusts. *n* = 12 biologically independent locusts. The data underlying the graphs shown in the figure can be found in S1 Data. **(b)** The semithin section of adult female ISM5. **(c)** The semithin section of adult male ISM5. **(d)** Tensile strength of ISM5 in adult female and male locusts. *n* ≥ 27 biologically independent locusts. The data underlying the graphs shown in the figure can be found in S1 Data. **(e–g)** The tensile strength, breaking strength and elongation at break of different ISMs (ISM3-ISM7) in adult female and male locusts. Student *t* test (two-tailed) was applied for two-group comparisons. Significant differences are denoted by **P < 0.01, ***P < 0.001, ****P < 0.0001. The data underlying the graphs shown in the figure can be found in S1 Data. **(h)** Structure diagram of female ISM modified according to Vincent (1981). **(i, i′)** The epicuticle of female ISM5 was observed by SEM. **(j)** The epicuticle of female ISM5 was observed by TEM. **(k)** The epidermal cells of female ISM5 were observed by TEM. **(l, l′)** The ultrastructure of female ISM5 was observed from longitudinal and transverse by TEM. Seg, Segment; ISM, Intersegmental membrane; PC, Pore canal; Epi, Epicuticle; Exo, Exocuticle; Endo, Endocuticle; EC, epidermal cells; Hl, Helix layer; El, Elastomer layer; PCF, Pore canal fiber; Bm, Basement membrane; Nu, Nucleus.

Adult female locusts lay their eggs in the soil by extending their abdomens, a process largely dependent on the extreme extensibility of the abdominal ISMs (S1A Fig and S1 Video). During oviposition, eggs are visible through the stretched ISMs, which retract to their normal state after egg-laying (S1B–S1F Fig). Comparative analysis revealed that the stretchable lengths of the female abdominal ISMs were significantly greater than those of the males (*t* = 10.355, *df* = 11, *p* < 0.0001) (Fig 1A and 1A′). Light microscope observation showed that in females, ISM4 (between Segs 4 and 5), ISM5 (between Segs 5 and 6, the widest), and ISM6 (between Segs 6 and 7) were wider than other ISMs (S2 Fig, upper), but the male ISMs showed no significant differences (S2 Fig, lower). Semithin section results further revealed a lamellar structure in females ISM5 that was distinct from the adjacent Seg5 (Fig 1B), while male ISM5 lacked this lamellar structure (Fig 1C). Importantly, mechanical testing showed that the male ISM5 had higher tensile strength than female ISM5, but endured for short duration (Fig 1D and S2 Video). We further compared the mechanical properties of abdominal ISMs (ISM3–7) between sexes, found that the male ISM3–7 exhibited greater tensile and breaking strength but significantly shorter elongation at break compared to the females (Fig 1E–1G). Specifically, the female ISM4–6 showed lower tensile and breaking strength but significantly greater elongation at break than other ISMs (Fig 1E–1G). However, the elongation at break of male ISM4–6 showed no significant difference from those of the other ISMs (Fig 1G). These results indicate that ISMs exhibit pronounced sexual dimorphism with ISM4–6 in adult females being highly extensible.

To investigate the ultra-structural basis of female ISM extensibility, we performed scanning electron microscopy (SEM) and transmission electron microscopy (TEM) analysis. SEM analysis revealed that ISM4–6 surfaces were highly folded, contrasting with the smooth and bristles surfaces of adjacent segments (Figs 1I, I′, S3A, S3A′, S4A, and S4B). TEM analysis further showed that ISM4–6 consisted of highly folded epicuticle, endocuticle, and epidermal cells, lacking the hard exocuticle observed in adjacent segments (Figs 1J, 1K, S3B, S3B′, S4C, and S4D). Moreover, the endocuticle in ISM4–6 was specialized into an elastomer layer (Fig 1L, 1L′, S3B, and S3B′). These findings demonstrate that adult female ISMs, particularly ISM4–6, exhibit remarkable mechanical extensibility, a trait absent in males, enabling their vital role in oviposition.

### Integrated transcriptomic and metabolic analysis reveals characteristics of female ISMs

**Fig 2:**
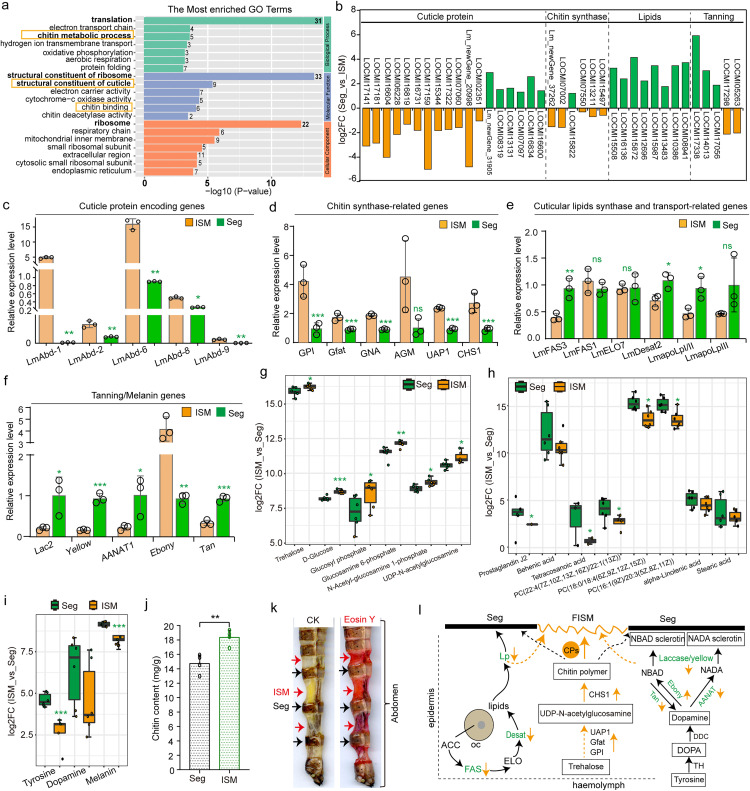
Differences in gene-expression and metabolic profile between segmental and intersegmental membranes. **(a)** GO enrichment of DEGs. Only GO terms with adjusted *P* < 0.01 are shown (Fisher’s exact test). The data underlying the graphs shown in the figure can be found in S1 Data. **(b)** DEGs (Seg_vs_ISM) involved in cuticle structure, chitin synthase, cuticle lipid and tanning are shown. **(c)** The expression level of DEGs involved in endocuticle structure (*LmAbd-1*, *LmAbd-2*, *LmAbd-6*, *LmAbd-8*, and *LmAbd-9*) were detected in Seg and ISM by RT-qPCR. **(d)** The expression level of DEGs involved in chitin synthase (*LmGPL*, *LmGfat*, *LmGNA*, *LmAGM*, *LmUAP1*, and *LmCHS1*) were detected in Seg and ISM by RT-qPCR. **(e)** The expression level of DEGs involved in cuticle lipid synthesis and transport pathways (*LmFAS3*, *LmELO*7, *LmDesat2*, *LmapoLpI/II*, and *LmapoLpIII*) were detected in Seg and ISM by RT-qPCR. **(f)** The expression level of DEGs involved in cuticle tanning-related enzymes (*LmLac2*, *LmAANAT1*, *LmADC*, *LmEbony*, and *LmTan*) were detected in Seg and ISM by RT-qPCR. Each circle represents a single individual. *n* = 3 biological replicates, and each target gene expression level was normalized to the expression of the internal reference gene *β-actin* (c–f). The data underlying the graphs shown in the figure can be found in S1 Data. **(g)** Comparison of chitin synthesis-related substances between Seg and ISM. **(h)** Comparison of lipid-related substances between Seg and ISM. **(i)** Comparison of tanning-related substances (Tyrosine, Dopamine, Melain) between Seg and ISM. Each dot represents a single individual. *n* = 6 biological replicates, and Boxplot showing the log2FC in the indicated metabolites measured by metabolomics (g–i). The data underlying the graphs shown in the figure can be found in S1 Data. **(j)** The difference in chitin content between Seg and ISM. Each circle represents a single individual. *n* = 4 biological replicates. Student *t t*est (two-tailed) was applied for two-group comparisons. Significant differences are denoted by *P < 0.05, **P < 0.01, ***P < 0.001 and ns, no significant difference. The data underlying the graphs shown in the figure can be found in S1 Data. **(k)** Stretching and Eosin staining of adult female locust abdomen. **(l)** Schematic diagram of structure formation difference between Seg and ISM. Seg and ISM represent segment and intersegmental membrane, respectively. FISM represents female intersegmental membrane.

To elucidate the molecular basis of different mechanical properties of ISMs, transcriptomic analysis were performed comparing ISM3–7 and their adjacent segments (Seg3–7) from adult female locusts at 2 days post-emergence, and approximately 59.8 million clean reads were obtained for each sample with an overall alignment rate of exceeding 91%. A total of 4,230 differentially expressed genes (DEGs, Seg_vs_ISM) were identified (FDR < 0.05, log2FC ≥ 1), and approximately 2,318 DEGs were highly expressed in female ISM3–7 (S5A Fig). Gene Ontology (GO) analysis revealed that these DEGs were significantly enriched in chitin metabolic process, structural constituent of cuticle, chitin binding, and other metabolism processes (adjusted *P* < 0.01, Fisher’s exact test) (Fig 2A and S1 Data). To further authenticate the gene-expression profiles, DEGs related to the cuticle were confirmed via real-time quantitative PCR (RT-qPCR). Consistent with the transcriptome data showing DEGs (Fig 2B and S1 Data), the transcription levels of structural CP genes involved in the formation of the endocuticle (*LmAbd1*, *LmAbd2*, *LmAbd6*, *LmAbd8*, and *LmAbd9*), especially two major structural CP genes, *LmAbd-1*, and *LmAbd-6*, were significantly greater in female ISM3–7 than in Seg3–7 (Figs 2C and S5B–S5C). Similarly, genes related to the chitin synthesis pathway, including *LmGPL*, *LmGfat*, *LmGNA*, *LmAGM*, *LmUAP*, and *LmCHS1*, showed elevated expression in female ISM3–7 than those in Seg3–7 (Figs 2D, S5B, and S5D). By contrast, the expression levels of genes related to cuticle lipid synthesis and transportation pathways (*LmFAS3*, *LmDesat-2*, *LmapoLpI/II*, and *LmapoLpIII*) in female ISM3–7 were significantly lower than those in Seg3–7 (Figs 2E, S5B, and S5E). Similarly, the expression levels of genes involved in the cuticle tanning pathway (*LmLac2*, *LmAANAT1*, *Lmyellow*, and *LmTan*) in female ISM3–7 were also significantly lower than those in Seg3–7 (Figs 2F, S5B and S5F).

To compare metabolic profiles with gene expression profiles between ISM3–7 and Seg3–7, we analyzed metabolomics in both positive and negative ion modes by using ultrahigh-performance liquid chromatography-high-resolution mass spectrometry (UPLC-MS). Orthogonal projections to latent structures-discriminant analysis (OPLS-DA) showed that the ISM and Seg samples were significantly separated into two categories, with metabolite intensity markedly higher in Seg3–7 (S6A and S6A′ Fig). There were significant differences in metabolite composition between ISM3–7 and Seg3–7 (S6B and S6B′ Fig), showing 329 up-regulated substances and 265 down-regulated substances in ISM3–7 relative to Seg3–7 (S6C and S6C′ Fig). Among these different metabolites, substances associated with the chitin synthesis pathway were enriched in ISM3–7 (Figs 2G and S7A), while those involved in cuticle lipid and tanning pathways were more abundant in Seg3–7 (Figs 2H–2L and S7B–S7C and S1 Data). Further assays confirmed chitin content was significantly higher in ISM3–7 than in Seg3–7 (*t* = −3.870, *df* = 3, *p* = 0.031) (Fig 2J), and eosin staining showed greater permeability in ISM3–7 (Fig 2K). The integrated transcriptomic and metabolomics analysis highlight that ISM3–7 possess extremely strong extensibility with weak barrier function, while Seg3–7 are specialized for the massive barrier function via cuticle tanning (Figs 2L and S7D).

### LmAbd-1 and LmAbd-6 control the formation of adult female ISMs

**Fig 3:**
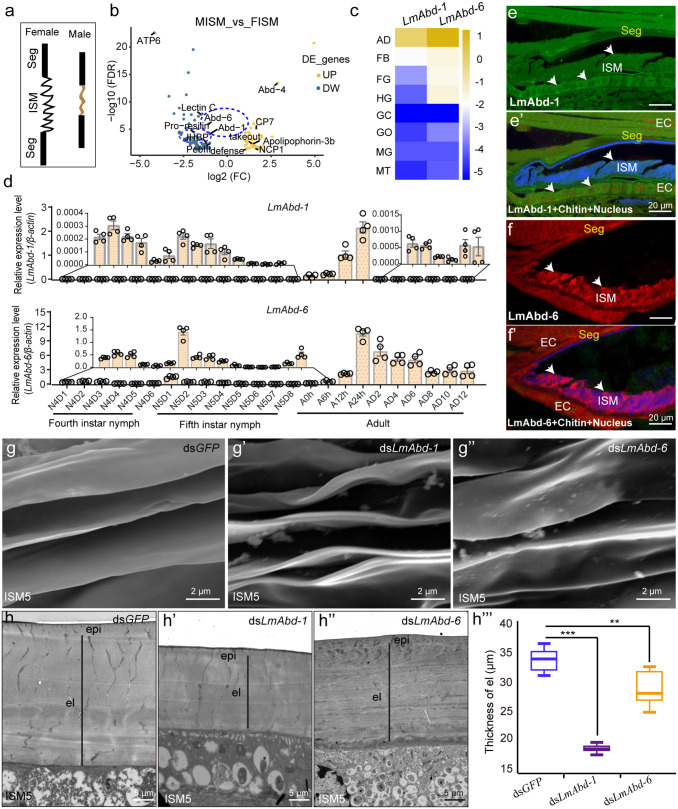
LmAbd-1 and LmAbd-6 are required for the structural formation of adult female ISMs. **(a)** Schematic diagram of female and male ISM. **(b)** The differentially expressed genes between adult female ISM (FISM) and male ISM (MISM) based on the transcriptomic data. The data underlying the graphs shown in the figure can be found in S1 Data. **(c)** The expression level of *LmAbd-1* and *LmAbd-6* in different tissues of adult female locusts was detected by RT-qPCR. AD, Abdomen; FB, Fat body; FG, Foregut; HG, Hindgut; GC, gastric caeca; GO, Gonad; MG, Midgut; MT, Malpighian tubules. The data underlying the graphs shown in the figure can be found in S1 Data. **(d)** Different stage-expression analyses of *LmAbd-1* and *LmAbd-6* from fourth-instar nymph to adult by RT-qPCR. N4D1-N4D6: Day 1 to day 6 of fourth-instar nymphs, N5D1-N5D8: Day 1 to day 8 of fifth-instar nymphs, A0h–A24h: 0h–24h after adult emergence, AD2–AD12: Day 2 to day 12 after adult emergence. *n* = 4 biological replicates. The data underlying the graphs shown in the figure can be found in S1 Data. **(e, e*****′***) Immunohistochemical localization of LmAbd-1 in female ISM5. The target protein LmAbd-1 is green (white arrow), the nucleus is red, and chitin is blue. **(f, f′)** Immunohistochemical localization of LmAbd-6 in female ISM5. The target protein LmAbd-6 is red (white arrow), the nucleus is green, and chitin is blue. EC: epidermal cells. **(g–g**″**)** The epicuticle of female ISM5 after *LmAbd-1* or *LmAbd-6* RNAi knockdown was observed by SEM. **(h–h**″**)** The ultrastructural of female ISM5 after knockdown of *LmAbd-1* or *LmAbd-6* was observed by TEM. Epi: Epicuticle, el: Elastomer layer. **(h′**″**)** The thickness of the elastomer layer was calculated after knockdown of *LmAbd-1* or *LmAbd-6*. *n* = 6 biological replicates. Student *t* test (two-tailed) was applied for two-group comparisons. Significant differences are denoted by **P < 0.01, and ***P < 0.001. The data underlying the graphs shown in the figure can be found in S1 Data.

To investigate the underlying mechanisms of sexual dimorphism in ISMs, we conducted transcriptome analysis between female ISM3−7 and male ISM3−7, and 119 DEGs were identified, of which 42 genes were upregulated and 77 genes were downregulated in females (Fig 3A and 3B and S1 Data). Notably, *LmAbd-1* and *LmAbd-6* were highly expressed in female ISMs, but not in male ISMs (S8A, S8A′, S8B, and S8B′ Fig). To determine whether and how the two major structural CP genes, *LmAbd-1* and *LmAbd-6*, are involved in the formation of adult female ISMs, we first detected their tissue expression specificity and found that both genes were highly expressed in the adult female abdomen but not in any other tissues (Fig 3C). The expression of *LmAbd-1* and *LmAbd-6* was low at each nymph stage (fourth- and fifth-instar nymphs), gradually increased after emergence until 24 h, and decreased thereafter; nevertheless, their expression levels were drastically greater in the early adult stage than in the nymph stages (Fig 3D). Furthermore, the expression levels of *LmAbd-1* and *LmAbd-6* were high in female ISM4-6, but low in the other detected female ISMs, which is consistent with the extreme extensibility of female ISM4−6 (S8C Fig). We then performed immunohistochemical localization of these two proteins in ISM5 and its adjacent Seg5, and the results showed that LmAbd-1 and LmAbd-6 were located in ISM5 and overlapped with chitin (Fig 3E, 3E′, 3F, and 3F′). The cDNA lengths of these two genes were 926 and 624 bp (containing three exons), encoding proteins with type 4 chitin-binding domains (ChtBD4), comprising 193 and 100 amino acids, respectively, with a RR-1 motif characteristic of CPs (S8D and S8E Fig). The alignment results revealed that the sequences of these two structural CPs were highly conserved in other insects, especially in the RR-1 motif (S9A–S9C Fig). Phylogenetic analysis indicated that despite their sequence similarity, Abd1 and Abd6 belong to distinct evolutionary branches (S10 Fig).

To further analyze their potential functions in adult female ISMs, RNAi was performed by injecting ds*LmAbd-1* or ds*LmAbd-6* into fifth-instar nymphs, using ds*GFP* as control. After knockdown of *LmAbd-1* or *LmAbd-6*, locusts developed successfully into adults (S11A–S11C Fig). However, SEM analysis of ISM4–6 at day 2 after adult emergence showed distorted and overlapping epicuticle in the RNAi-treated individuals compared to the control (Figs 3G–3G″ and S12A). TEM results further demonstrated that the elastomer layer in the endocuticle of ISM4–6 was significantly thinner in the RNAi-treated locusts, leading to reduced cuticle layers (Figs 3H–3H′″ and S12B). These results indicate that LmAbd-1 and LmAbd-6 tightly control the formation of adult female ISMs.

### *LmAbd-1* and *LmAbd-6* control the extensibility of adult female ISMs and oviposition behavior

**Fig 4:**
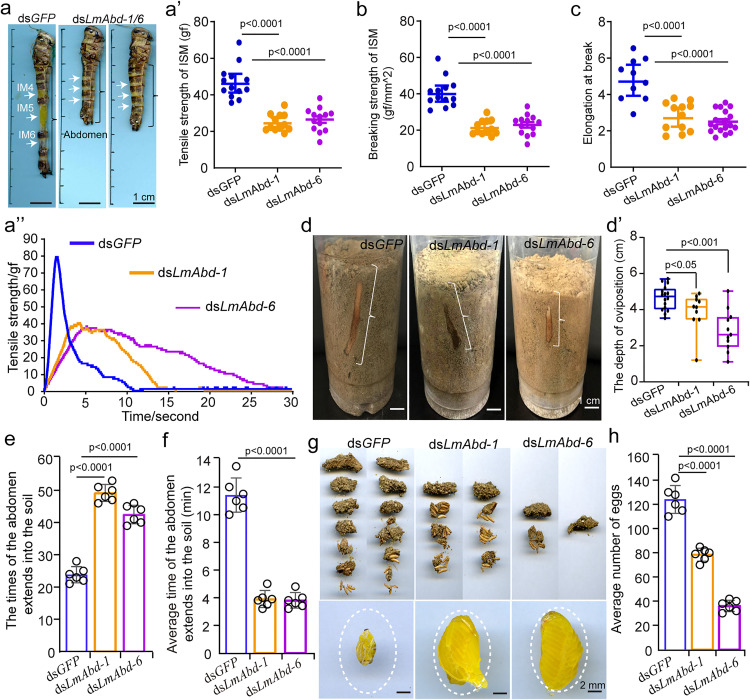
LmAbd-1 and LmAbd-6 are crucial for the extensibility of adult female ISMs and oviposition behavior. **(a, a**″**)** Stretching and tensile strength of female abdominal ISMs after injection of ds*GFP*, ds*LmAbd-1* or ds*LmAbd-6*, respectively. The data underlying the graphs shown in the figure can be found in S1 Data. **(b, c)** Breaking strength and elongation at break of female ISM5 after injection of ds*GFP*, ds*LmAbd-1* or ds*LmAbd-6*, respectively. n ≥ 13 biologically independent locusts (a′–c). The data underlying the graphs shown in the figure can be found in S1 Data. **(d, d′)** Observation and quantification of the depth of the abdominal stretch in the soil of the adult female locusts during oviposition after injection of ds*GFP*, ds*LmAbd-1* or ds*LmAbd-6*, respectively. *n* ≥ 7 biologically independent locusts. The data underlying the graphs shown in the figure can be found in S1 Data. **(e, f)** The quantitative statistics of the times and the average time of laying eggs in the soil of the adult female locusts. The data underlying the graphs shown in the figure can be found in S1 Data. **(g, h)** The number of oocysts and eggs was calculated, and the ovary size of female locusts was observed after oviposition. *n* = 6 biologically independent locusts **(e–h)**. The data underlying the graphs shown in the figure can be found in S1 Data. Student *t* test (two-tailed) was applied for two-group comparisons. The data are shown as the mean ± SEM.

To determine whether and how the absence of *LmAbd-1* or *LmAbd-6* affects the extensibility of adult female ISMs and oviposition behavior in locusts, we first detected the mechanical properties of female ISMs following RNAi-mediated knockdown of *LmAbd-1* or *LmAbd-6*. In both RNAi groups, the abdominal stretch length of female locusts was reduced by approximately 50% compared to the control group (Fig 4A). Similarly, the tensile strength of ISM5 decreased by half in both RNAi treatment groups (*t* = 7.921, *df* = 11, *p* < 0.0001; *t* = 8.788, *df* = 12, *p* < 0.0001) (Fig 4A′ and 4A″), wi*t*h breaking strength and elongation at break reduced by 50% in both groups (*t* = 4.406, *df* = 9, *p* = 0.002; *t* = 4.637, *df* = 9, *p* = 0.001) (Fig 4B and 4C; S3 and S4 Videos). The dep*t*h of *t*he abdominal insertion into the soil during oviposition was also affected. As shown in Fig 4D and 4D′, the depth in the *LmAbd-1* RNAi treatment group decreased only 10% compared to the control group (*t* = 2.365, *df* = 9, *p* = 0.042), while it decreased by half in *t*he *LmAbd-6* RNAi treatment group (*t* = 5.059, *df* = 10, *p* < 0.001). Moreover, the frequency of egg laying or a*t*tempting to lay eggs in the soil increased by half in both RNAi treatment groups (*t* = −24.033, *df* = 5, *p* < 0.0001; *t* = −18.265, *df* = 5, *p* < 0.0001); while the average dura*t*ion of oviposition decreased by approxima*t*ely two-thirds (*t* = 20.117, *df* = 5, *p* < 0.0001; *t = *21.664, *df* = 5, *p* < 0.0001) (Fig 4E and 4F). Furthermore, examination revealed tha*t* the number of oocysts laid in both RNAi treatment groups was significantly lower than in the control group (Fig 4G); dissected ovaries from the RNAi-treated female locusts were markedly larger than in the control group (Fig 4G and S13A–S13A″). Importantly, the average number of laid eggs decreased by approximately one-third in the *LmAbd-1* RNAi treatment group and by two-thirds in the *LmAbd-6* RNAi treatment group (*t* = 8.406, *df* = 5, *p* < 0.001; *t = *24.923, *df* = 5, *p* < 0.0001) (Figs 4H and S13B–S13B″). These results indicate that *LmAbd-1* and *LmAbd-6* control *t*he formation of adult female ISMs, and consequently, the extensibility of female ISMs and the ISM-dependent oviposition behavior.

### LmJHBP-mediated JH signaling promotes the expression of *LmAbd-1* and *LmAbd-6*

**Fig 5:**
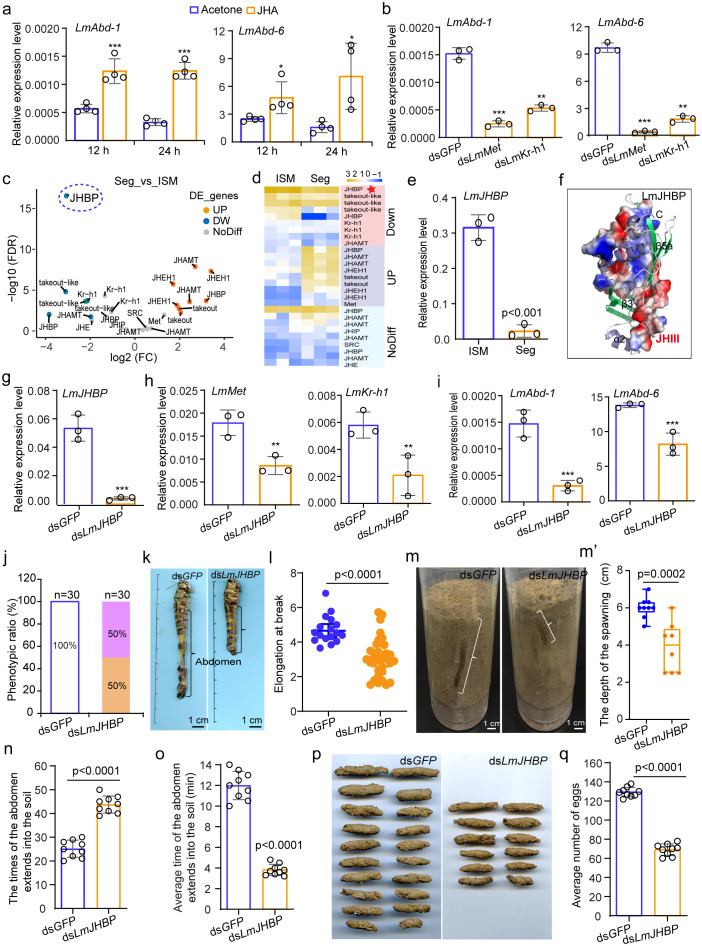
The distinctive extensibility of adult female ISMs is regulated by JHBP-mediated JH signaling. **(a)** The expression of *LmAbd-1* and *LmAbd-6* were induced by JHA. *n* = 4 biological replicates. **(b)** The expression of *LmAbd-1* and *LmAbd-6* were detected after injection of ds*GFP*, ds*LmMet*, or ds*LmKr-h1*, respectively. *n* = 3 biological replicates. **(c)** Volcano map of differentially expressed genes about the JH signal between Seg and ISM. **(d)** Heat map representing gene-expression levels is involved in the JH signal. Heat-map signal indicates log2-fold change value relative to the mean expression level within the group. The red signal represents higher expression, whereas blue represents lower expression. Seg and ISM represent segment and intersegmental membrane, respectively. **(e)** The expression level of *LmJHBP* in Seg and ISM. **(f)** Structure pattern diagram of LmJHBP protein binding to JHIII. **(g)** The expression level of *LmJHBP* after injection of ds*LmJHBP*. **(h)** The expression level of *LmMet* and *LmKr-h1* after knockdown of *LmJHBP*. **(i)** The expression level of *LmAbd-1* and *LmAbd-6* after knockdown of *LmJHBP*. *n* = 3 biological replicates (e–i). **(j)** The phenotype of locust after *LmJHBP* knockdown. **(k)** Abdominal stretching lengths after knockdown of *LmJHBP*. **(l)** Elongation at break after knockdown of *LmJHBP*. *n* ≥ 17 biologically independent locusts. **(m, m′)** Observation and quantification of the depth of spawning after knockdown of *LmJHBP*. **(n, o)** The quantitative statistics of the times and the average time of laying eggs in the soil after knockdown of *LmJHBP*. **(p, q)** The image of the ovary size of locusts and the number of oocysts after spawning. *n* ≥ 8 biologically independent locusts (m–q). Student *t* test (two-tailed) was applied for two-group comparisons. The data are shown as the mean ± SEM. *P < 0.05, **P < 0.01, and ***P < 0.001. ns: no significant difference. The data underlying the graphs show in the figure can be found in S1 Data.

Previous study have suggested that JH influences the mechanical properties of ISMs in locusts after the corpora allata are removed from immature adult females [14]. Interestingly, the expression levels of *LmAbd-1* and *LmAbd-6* in adult females resemble the fluctuation in JH titers [29]. We thus wondered whether and how JH exerts its effect by regulating the expression levels of *LmAbd-1* and *LmAbd-6*, either directly or indirectly. After induction with the JH analogue (JHA) by topical application on day 2 of adult female locusts, we found that the expression levels of *LmAbd-1* and *LmAbd-6* were significantly up-regulated at 12 h and further increased at 24 h (Fig 5A). To verify the regulation of *LmAbd-1* and *LmAbd-6* by JH signaling, RNAi knockdown of the JH receptor gene *LmMet* and the key JH downstream transcription factor *LmKr-h1* was performed. The results showed that the expression of *LmAbd-1* and *LmAbd-6* was significantly decreased after *LmMet* and *LmKr-h1* RNAi treatments compared with the control (Fig 5B). Further analysis of *LmKr-h1* expression in female ISMs revealed high level in ISM5, consistent with the expression pattern of *LmAbd-1* and *LmAbd-6* (S14A Fig). Furthermore, in the *LmKr-h1* RNAi treatment group, the female ISM5 was narrower than in the control (S14B Fig), and the tensile strength, breaking strength, and elongation at break were all significantly decreased (*t* = 3.002, *df* = 18, *p* = 0.008; *t* = 2.159, *df* = 18, *p* = 0.045) (S14C–S14C″ Fig). We further compared the transcriptomics data from ISM3–7 and Seg3–7, and found that genes related to JH signaling, including *Kr-h1* and the JH binding protein gene *JHBP*, were upregulated in female ISMs (Fig 5C, 5D and S1 Data). The results together indicate that the JH signaling induces the expression of *LmAbd-1* and *LmAbd-6* in female adult ISMs.

To further investigate the effect of JH signaling on gene expression and the extensibility of ISM, we topically applied JHA to adult males on day 2 post-emergence. Our analysis revealed a significant increase in the expression levels of *LmAbd-1* and *LmAbd-6* at 6 h post-treatment (S15A Fig). Concurrently, the width of male ISM5 was significantly greater compared to the control group (*t* = −1.756, *df* = 3, *p* = 0.033) (S15B and S15B′ Fig). Notably, both tensile strength and breaking strength were significantly reduced (*t* = 2.204, *df* = 18, *p* = 0.041) (S15C and S15C′ Fig), whereas the elongation at break were significantly increased (*t* = −4.008, *df* = 18, *p* = 0.001) (S15C″ Fig). These findings further support the conclusion that JH signaling enhances ISM extensibility by upregulating the expression of *LmAbd-1* and *LmAbd-6*.

Notably, we discovered that *LmJHBP* was highly expressed in female ISMs compared to female Segs and male ISMs, with particularly pronounced expression observed female ISM4-6 (*t*=19.885, *df*=2, *p*=0.003) (Figs 3B, 5E, S16A, and S16B and S1 Data). Homology analysis revealed that the JH-binding domain of LmJHBP is highly conserved across species (S16C and S16D Fig). Homology modeling of LmJHBP was carried out by using BmJHBP from *Bombyx mori* as an appropriate template [30], and the results showed that LmJHBP and BmJHBP share a similar protein structure with three α-helical (α1–α3) structures and five β-lamellar (β1–β5) structures (S17A and S17B Fig), forming a JHIII-binding region (Figs 5F, S17C, and S17D). We then wondered whether and how LmJHBP mediates JH signaling in ISM4-6. We blocked the JH signaling by RNAi knockdown of *LmMet* and *LmKr-h1*, and found that the expression level of *LmJHBP* did not significantly change (S18A and S18B Fig). Conversely, the expression levels of *LmMet* and *LmKr-h1* were significantly downregulated after *LmJHBP* RNAi treatments (*t* = 6.214, *df* = 2, *p* = 0.025; *t* = 6.803, *df* = 2, *p* = 0.021) (Fig 5G and 5H), which is consistent with the decreased expression levels of *LmAbd-1* and *LmAbd-6* (*t* = 8.158, *df* = 2, *p* = 0.015; *t* = 6.401, *df* = 2, *p* = 0.024) (Fig 5I). These results indicate that LmJHBP is indispensable for normal JH signaling that promotes the expression of *LmAbd-1* and *LmAbd-6*, likely controlling the sensitivity to JH particularly in ISM4-6.

It was of interest to further determine whether LmJHBP regulates the extensibility of female ISMs and oviposition behavior through JH signaling. After *LmJHBP* RNAi knockdown in fifth-instar nymphs, approximately 50% of locusts normally molt to adults (Fig 5J), and their abdominal stretch length was reduced compared to the control (Fig 5K). Although the tensile and break strength of ISM5 did not differ significantly between the ds*LmJHBP*-treated and control locusts (S18C, S18C′, and S18D Figs), the elongation at break of ISM5 was decreased by approximately half in the ds*LmJHBP*-treated group (*t* = 2.852, *df* = 16, *p* < 0.0001) (Fig 5L and S5 Video). Similar to the results of *LmAbd-1* and *LmAbd-6* RNAi knockdown, the depth of abdominal stretch during oviposition of adult female locusts was reduced by one-third after *LmJHBP* RNAi knockdown (*t* = 4.475, *df* = 7, *p* = 0.0002) (Fig 5M and 5M′). The *t*imes that the abdomen extended into the soil to oviposit or attempt to oviposit increased by approximately 50% (*t* = −9.569, *df* = 8, *p* < 0.0001) (Fig 5N), bu*t* the average time at which the abdomen extended into the soil was shortened by two-thirds (*t* = 19.461, *df* = 8, *p* < 0.0001) (Fig 5O). At *t*he end of oviposition, the remaining ovaries in the ds*LmJHBP*-treated group were much larger than those in the control group (S19 Fig). In contrast, the number of oocysts and eggs in the ds*LmJHBP*-treated group was reduced by approximately half (*t* = 19.751, *df* = 8, *p* < 0.0001) (Fig 5P and 5Q). These results indicate *t*hat the LmJHBP-mediated JH signaling is crucial for regulating the extensibility of adult female ISMs and oviposition behavior through the modulation of *LmAbd-1* and *LmAbd-6* expression*.*

### Sex differentiation genes regulate the expression of *LmJHBP* and *LmAbd-1/6* to control ISM extensibility

**Fig 6:**
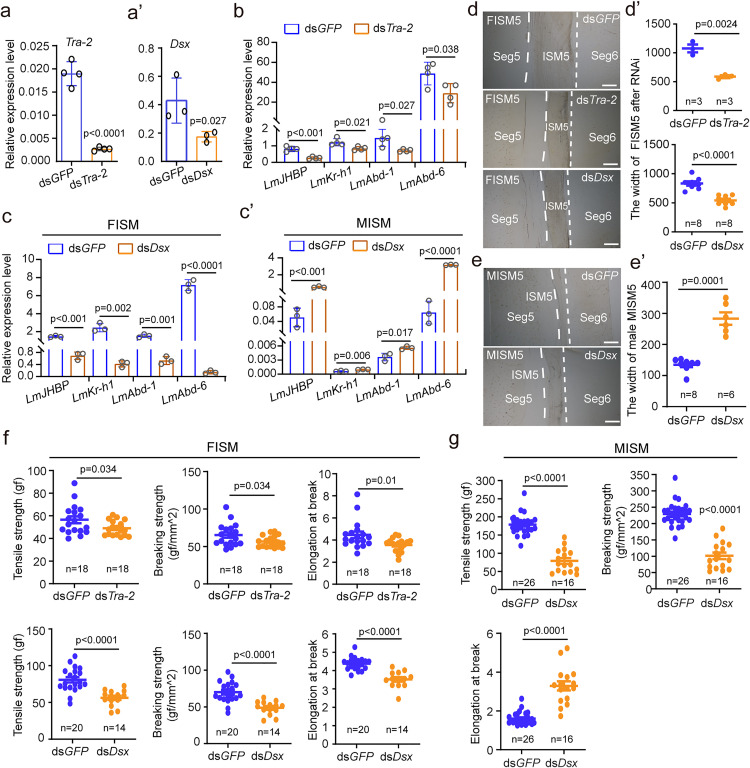
The sex differentiation pathway controls the extensibility of ISMs. **(a)** The expression of *Tra-2* in adult female ISMs by RT-qPCR after knockdown of *Tra-2*. *n* = 4 biological replicates. **(a′)** The expression of *Dsx* in adult female ISMs by RT-qPCR after knockdown of *Dsx*. *n* = 3 biological replicates. **(b, c)** The expression of *LmJHBP, Kr-h1*, *LmAbd-6*, and *LmAbd-1* in adult female ISMs by RT-qPCR after knockdown of *Tra-2* and *Dsx*, respectively. **(c′)** The expression of *LmJHBP, Kr-h1*, *LmAbd-6*, and *LmAbd-1* in adult male ISMs by RT-qPCR after knockdown of *Dsx*. **(d, d′)** The microstructure difference (width) of female intersegment membrane 5 (ISM5) between *Tra-2* or *Dsx* RNAi treatment and control groups. **(e, e′)** The microstructure difference (width) of male intersegment membrane 5 (ISM5) between *Dsx* RNAi treatment and control groups. **(f)** The tensile strength, breaking strength and elongation at break of female ISM5 after knockdown of *Tra-2* and *Dsx*, respectively. *n* ≥ 14 biologically independent locusts. Student *t* test (two-tailed) was applied for other two-group comparisons. The data are shown as the mean ± SEM. **(g)** The tensile strength, breaking strength and elongation at break of male ISM5 after knockdown of *Dsx*. *n* ≥ 16 biologically independent locusts. Student *t* test (two-tailed) was applied for other two-group comparisons. The data are shown as the mean ± SEM. The data underlying the graphs shown in the figure can be found in S1 Data. FISM and MISM represent female and male intersegmental membrane, respectively.

To further explore the underlying mechanisms of sexual dimorphism in ISMs, we also identified two key genes in the sex differentiation pathway, *transformer 2* (*Tra-2*) and *doublesex* (*Dsx*), from the transcriptomics data. Importantly, RNAi knockdown of *Tra-2* or *Dsx* in adult females significantly reduced the expression of *LmJHBP*, *LmKr-h1*, *LmAbd-1* and *LmAbd-6* (Fig 6A, 6A′, 6B, and 6C). Meanwhile, we knocked down *Dsx* expression in adult males and found that the mRNA levels of *LmJHBP*, *LmKr-h1*, *LmAbd-1*, and *LmAbd-6* were significantly increased compared to those of the control (Fig 6C′). Furthermore, the ISM4–6 in the *Tra-2* or *Dsx* RNAi-treated adult females was narrower than in the control (*t* = 6.292, *df* = 2, *p* = 0.0024; *t* = 5.468, *df* = 7, *p* < 0.0001) (Figs 6D, 6D′, and S20A), whereas the male ISM4–6 in *Dsx* RNAi treatment group was wider than that of control (*t* = −5.852, *df* = 5, *p* < 0.0001) (Figs 6E, 6E′, and S20B). Correspondingly, *t*he mechanical properties of ISM5 such as tensile strength, breaking strength, and elongation at break were significantly decreased in each RNAi treatment group (Fig 6F). In males, the tensile strength and breaking strength of ISM5 were decreased (*t* = 12.451, *df* = 15, *p* < 0.0001), whereas elongation a*t* break was significantly increased in the RNAi treatment group (*t* = −6.178, *df* = 15, *p* < 0.0001) (Fig 6G). The results show tha*t* forming a Tra/Dsx-JHBP axis in females, the sex differentiation pathway (*Tra-2* and *Dsx*) lies upstream of the *LmJHBP*-mediated JH signaling to induce *LmAbd-1* and *LmAbd-6* expression, and consequently controlling the extensibility of ISMs in adult female locusts.

## Discussion

In this study, we revealed that the high flexibility of abdominal ISMs in adult female locusts are determined by two major ISM proteins (LmAbd-1 and LmAbd-6) which are critical for enabling oviposition behavior. Moreover, the female-specific expression of *LmAbd-1* and *LmAbd-6* is controlled by the Tra/Dsx-JHBP axis and thus JH signaling, determining the corresponding sexual dimorphism in a stage-specific manner.

### LmAbd-1 and LmAbd-6 determine adult female ISMs extensibility and oviposition behavior

Our results showed distinctly sexual dimorphism in the abdominal ISMs of the migratory locust, aligning with previous studies [14,16]. It is of note that the extensibility of female ISMs (particularly ISM4–6) is superior to that of male ISMs due to structural and morphological differences. We discovered that the increased extensibility and expansive nature of ISM4–6 in adult female locusts are inversely proportional to the narrow surfaces area and rigidity of other inextensible female ISMs such as ISM2–3. Meanwhile, this dimorphism extends to the female abdominal ISMs and their adjacent Segs. Previous research has highlighted that the mechanical properties of the insect cuticle are influenced by chitin architecture, degree of sclerotization, and hydration [31], but little is known about the precise combination of structural CPs within the cuticular matrix in determining the properties [32]. Our study revealed significant compositional differences between ISMs and Segs, particularly in chitin and structural CPs. Segs predominantly contain enzymes and substances required for cuticle tanning, reflecting their rigid barrier function. In contrast, ISMs lack a hard exocuticle and are characterized by a resilient endocuticle (elastomer layer), which is accentuated by intricate cross-links between structural CPs (i.e., LmAbd-1 and LmAbd-6) and chitin fibers. This unique molecular composition enables ISMs to demonstrate exceptional flexibility, embodying the strength of adaptability in their structural design.

Structural CPs are ubiquitous across insect species [33–35]. Here, we uncovered a previously unrecognized function for two major structural CPs, LmAbd-1 and LmAbd-6, specifically expressed in the abdominal ISM4−6 of adult females. Both proteins belong to the RR-1 subfamily of CPR family, which is a group of proteins reported to be used for specific soft, flexible cuticles such as ISMs [36]. Moreover, the two proteins display an astonishing degree of conservation, highlighting the striking similarities in different locust species [37]. Knockdown of their encoding genes results in structural defects in female ISMs, which lead to decreased extensibility of ISMs and abnormal oviposition behavior. Therefore, our study reveals a previously unrecognized role of structural CPs in the formation of sexually dimorphic ISMs and egg-laying strategies.

### The expression of *LmAbd-1* and *LmAbd-6* is controlled by the Tra/Dsx-JHBP axis and thus JH signaling

To elucidate the regulatory mechanisms underlying the expression of *LmAbd-1* and *LmAbd-6*, we examined the role of JH signaling. The majority of proteins are deposited in ISMs before sexual maturation, and the transformation from inextensible to highly extensible ISM depends on an increased titer of JH [14]. We first revealed that the highly expressed *LmJHBP* in female ISM4–6 is crucial for the ISM extensibility and oviposition behavior, resembling the roles of *LmAbd-1* and *LmAbd-6*. Then, we showed that JHBP mediated JH signaling for this regulation, likely via regulating the JH sensitivity specifically in female ISM4–6.

**Fig 7:**
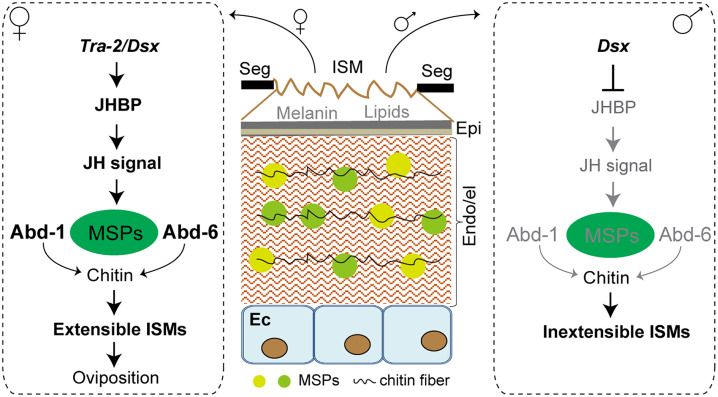
The Tra/Dsx-JHBP axis controls the extensibility of ISMs by regulating major structure proteins in locusts. The Tra/Dsx-JHBP axis regulates two key proteins to control ISM extreme extensibility that display a sexual dimorphism in adult female and male locusts. MSP: major structural protein. Seg, Segment; ISM, Intersegmental membrane; Epi, Epicuticle; Endo, Endocuticle; el, Elastomer layer; Ec, Epidermal cells.

Intriguingly, there are discernible differences in the proteins of the abdominal cuticle between male and female desert locusts, particularly within sexually dimorphic ISMs [37], although the role of these proteins were unknown. We found significant sex-biased enrichment of the *LmJHBP* and *LmAbd-1/6* transcripts, which were specifically in female ISMs but not in males. The sex differentiation pathway is a conserved switch or regulator that governs a set of downstream genes that direct sexually dimorphic traits [38,39]. In *Caenorhabditis elegans* and *D. melanogaster*, sex-specific gene expression is mediated by the transcriptional effectors of their sex-determination pathways [40–41]. In our investigation, we revealed *tra-2* and *Dsx*, two key genes regulated by the original signal of sex determination [42–44], regulate the expression of *LmJHBP* exclusively in adult female ISMs. Thus, we discovered the Tra/Dsx-JHBP axis, which mediates JH signaling in adult female ISMs and thus *LmAbd-1* and *LmAbd-6* expression. While in adult male ISMs, their expression was inhibited by *Dsx*. Together with studies on other insects in which sex differentiation and hormone regulators control sex-specific characteristics, it appears that the coordinated regulation of sex-specific characteristics by sex differentiation and hormone signaling could be universal across insects [45–48]. Thus, we elucidated the dynamics of extensible ISM formation in adult female locusts and their oviposition behavior, establishing a link between adult female ISM extensibility and male ISM inextensibility through the Tra/Dsx-JHBP axis and thus JH signaling in two major ISM genes (Fig 7).

The adaptability of the ISMs, controlled by the Tra/Dsx-JHBP axis, exemplifies a remarkable evolutionary innovation that enables females to optimize their reproductive success in challenging environments. Drawing parallels to the well-characterized mechanisms of segment formation and sexually dimorphic traits in *Drosophila* [49], we propose such an adaptive mechanism has evolved in locusts specifically for oviposition in soils. This adaptation stands in contrast to the oviposition strategies of other insects, such as mosquitoes, which lay eggs on water surfaces [50]. The locust’s ability to penetrate and oviposit in soils highlights the remarkable diversity of reproductive adaptations across insect taxa, shaped by the interplay between genetic regulation, mechanical properties, and ecological constraints. By elucidating the molecular and mechanical underpinnings of this adaptation, our work significantly enriches the understanding of sexual dimorphism, behavioral adaptability, and the evolutionary dynamics of segmental specialization in insects.

## Methods

### Insects

Eggs of *L. migratoria* maintained in our laboratory were placed in a perforated transparent plastic bag and incubated in a constant temperature incubator. After hatching into the first instar nymphs were transferred to a hand-stitched sarong with a size of about 30 cm × 30 cm × 30 cm, and reared under controlled conditions: the temperature at 30 ± 2 °C, the relative humidity at 40 ± 10%, and the photoperiod of 14 h:10 h (L:D). Nymphs were fed fresh wheat seedlings and bran ad libitum.

### Semithin section and observation of light microscope

ISM5 and its adjacent Seg were dissected from adult female and male locusts at 2 days after emergence. The structural features were observed by semithin section analysis as previously described [51]. To observe the morphology of ISM, the female and male ISMs from segments two to eight (ISM2 to ISM7) were dissected and observed under a light microscope (CNOPTEC, Chongqing, China).

### RT-qPCR

To explore the tissue-specific expression of *LmAbd1* and *LmAbd6*, eight different tissues were dissected from female adults (day 1), including abdomen (AD), Fat body (FB), Foregut (FG), Hindgut (HG), gastric caeca (GC), Gonad (GO), Midgut (MG), and Malpighian tubules (MT). For the temporal expression of *LmAbd1* and *LmAbd6*, the ADs from fourth-instar nymph to adult were prepared. Different ISMs (ISM2-ISM7) from female adults were dissected for detecting the expression of *LmAbd1*, *LmAbd6*, *LmJHBP* and *LmKr-h1*. All samples were collected with at least three biological replicates and each with four nymphs or adults and frozen in liquid nitrogen. Total RNA of all samples were extracted by RNAiso Plus extraction reagent (TaKaRa, Kusatsu, Japan), and detected by 1.5% agarose gel electrophoresis to check the quality. The total RNAs were quantified using a Nanodrop 2000, and then one μg of total RNA as a template was used to synthesize cDNA according to the instructions of reverse-transcription reagents (TaKaRa, Japan). Each cDNA sample was diluted 10-fold for reverse transcription quantitative PCR (RT-qPCR) analysis. Relative mRNA expression levels were quantified with the FastStart Universal SYBR Green Master Mix Kit (Toyobo Co., Ltd, Osaka, Japan) and Bio-Rad CFX Connect Real-Time System (Bio-Rad, Hercules, CA, USA). The RT-qPCR reactions contained 10 μL of 2 × SYBR Premix EX Taq (TaKaRa, Japan), 0.4 μL of 50 × ROX Reference Dye (TaKaRa, Japan), and 2 μL of specific primers (2 μM), and consisted of initial step at 95 °C for 30 s, followed by 40 cycles of 95 °C for 5 s and 60 °C for 31 s. Target gene expression level was calculated with the 2^−ΔΔ*Ct*^ method and normalized to the expression of the internal reference gene *β-actin* (KX276642). The primers used for RT-qPCR in this study are listed in S1 Table.

### RNA interference

For RNAi experiment, the cDNA sequences of target genes (*LmAbd-1*, *LmAbd-6*, *LmJHBP*, *LmMet*, *LmKr-h1*, *Tra-2*, and *Dsx*) retrieved from our transcripomic data (GEZB00000000 and PRJNA1121037). Double-stranded RNA (dsRNA) of *GFP* and target genes were synthesized in vitro through the T7 RiboMAX Express RNAi System (Promega, Madison, WI, USA), following the manufacturer’s standardized protocol. The dsRNA synthesis was performed using sequence-specific primers designed for each target gene. Approximately 10 μg (2 μg/μL) of dsRNA was injected into the hemocoel between the second and third abdominal segments of each fifth-instar nymph (day 2 or day 5) by using a microinjector (Ningbo, China). The ds*GFP*-injected group served as the control. After 48 h of treatment, the silencing efficiency of target genes was detected by RT-qPCR as described above. The primers for target genes dsRNA synthesis are shown in S1 Table.

### Scanning electron microscopy and transmission electron microscopy

ISMs and Segs from adult female locusts, as well as ISMs from the dsRNA-treated and control groups were collected at 2 days after emergence for structural analysis using SEM and TEM analysis, respectively. SEM analysis was performed as previously described [51]. The samples were fixed overnight at 4°C in a 3% glutaraldehyde solution for TEM analysis. Ultrathin sections (80 nm) were prepared following established protocols [23]. The images were captured using a JEM-1200EX transmission electron microscope (JEOL, Tokyo, Japan).

### RNA-seq

The ISMs and their adjacent Segs from abdominal segments 3–7 were dissected from adult female and male locusts at 2 days post-emergence. Total RNA was extracted using TRIzol reagent (Invitrogen) from the ISMs and Segs of abdominal segments 3–7 in adult female locusts, as well as from the ISMs of segments 3–7 in adult males. For each group, three biological replicates were prepared, with each replicate consisting of five individuals. The mRNAs in each sample were enriched and complementary cDNA libraries were generated by Biomarker Technologies (Beijing, China), and sequenced on the Illumina HiSeq2000 platform. Raw sequencing reads were filtered using SOAPnuke software to remove adaptor-contaminated sequences and reads with a low-quality base ratio. High-quality cleaned reads were obtained and stored in fastq format, and then assembled using Trinity short read assembly to produce unigenes, which were subsequently mapped to the locust genome sequence (AVCP00000000) using high-throughput spliced aligner HISAT2 software. The assembled ungenes were compared with those in the NR, NT, SwissProt, COG and KEGG databases by BLAST, and the cut-off E-value was 10^−5^. HTSeq software was used for calculating the unique mapped read counts, and single gene abundance was quantified as fragments per kilobase of transcript per million reads (FPKM), and subsequently analyzed for DEGs by DESeq2 software based on the cutoff criteria of log2(fold change)>1 and adjusted *P*-value <0.05 in each comparison. The fastq files of the transcriptome sequence are available at BioProject PRJNA1121037.

### Metabolomics analysis

Approximately 50 mg of ISMs and Segs from the abdomen of adult female locusts (2 days post-emergence) were homogenized in 1,000 µL of extraction solvent (methanol: acetonitrile: water = 2:2:1, v/v) containing an internal standard (IS; final concentration: 2 mg/L, solvent-to-IS ratio 1000:2). Six biological replicates (each consisting of at least five locusts) were processed. Samples were vortexed for 30 s, mixed with ceramic beads, and ground at 45 Hz for 10 min, followed by sonication in an ice-water bath for 10 min and incubation at −20 °C for 1 h. After centrifugation (12,000 rpm, 15 min, 4 °C), 500 µL of supernatant was collected, dried under vacuum, and reconstituted in 160 µL of acetonitrile: water (1:1, v/v). The solution was vortexed (30 s), sonicated (10 min, ice-water bath), and centrifuged again (12,000 rpm, 15 min, 4 °C). A 120 µL aliquot of the final supernatant was transferred to a 2 mL injection vial. For quality control, 10 µL from each sample was pooled. Liquid chromatography–mass spectrometry (LC-MS) analysis was conducted using a Waters Acquity I-Class PLUS UPLC system coupled to a Waters Xevo G2-XS QToF high-resolution mass spectrometer. Separation was achieved with a Waters Acquity UPLC HSS T3 column (1.8 µm, 2.1 × 100 mm) at 40 °C. The mobile phase comprised 0.1% formic acid in water (A) and 0.1% formic acid in acetonitrile (B) for both positive and negative ionization modes. The injection volume was 1 µL.

Mass spectrometry parameters included MSe acquisition mode (MassLynx V4.2 software) with alternating low (2 V) and high (10–40 V) collision energies at 0.2 s/spectrum. Electrospray ionization (ESI) conditions were: capillary voltage ±2,000 V (positive/negative mode), cone voltage 30 V, ion source temperature 150 °C, desolvation temperature 500 °C, cone gas flow 50 L/h, and desolvation gas flow 800 L/h. Raw data were processed using Progenesis QI software (Waters Corporation) for peak extraction, peak annotation, and alignment. The METLIN database and a custom Biomarker Technologies Platform (Beijing, China) database were utilized for metabolite identification, with theoretical fragment matching and mass accuracy thresholds set to ≤100 ppm. The data involving the sample name, peak number and normalized peak area were analyzed using R package ropls (version 3.3.2) for principal component analysis (PCA) and orthogonal projections to latent structures-discriminate analysis (OPLS-DA). Metabolites with variable importance in the projection values > 1, Fold change > 1.5, and adjusted *P*-value < 0.05 were considered differentiated.

### Chitin content determination

The fourth and sixth abdominal ISMs and Segs from adult female locusts (four samples with at least 15 individuals per sample, respectively) at 2 days after emergence were dissected to determine the chitin content. Each sample was dried at 90 °C until the weight was stable. Chitin extraction and content measurement were performed as previously described [52]. Glucosamine was selected to prepare the standard curve. The chitin content was calculated as follows: chitin (mg)/tissue weight (g) = glucosamine concentration (μg/mL)/tissue weight (g).

### Eosin Y Staining

To investigate permeability differences between ISMs and Segs of female adults at day 2 after emergence, Eosin Y staining was performed. After the abdomen of the adults was stretched, the adults were transferred to a 10 mL centrifuge tube containing 10 mL of dye solution (0.5% Eosin (W/V, Sigma, a red water-soluble dye with a molecular mass of 691.86 Da)), incubated at 45 °C for 30 min, and then washed three times with water as previously described [53]. Finally, the locusts were photographed with an Epson Perfection V700 photo using Epson Scan software.

### Sequence and phylogenetic analysis

The cDNA sequences of the target genes were obtained from the whole body transcriptomic database (GEZB00000000). The reading frames of genes were analyzed at NCBI (https://www.ncbi.nlm.nih.gov). Protein signal peptide and structural domain were predicted using SignaIP 5.0 server (http://www.cbs.dtu.dk/services/SignalP/) and SMART (http://smart.embl-heidelberg.de/), respectively. The protein sequences were analyzed using the MEGA 7 software to construct a phylogenetic tree by adopting the neighbor-joining (NJ) method with 1,000 bootstrap replicates (50% cut-off). The GenBank accession numbers of protein sequences from the different species used to construct the phylogenetic tree are listed in S2 Table.

### Polyclonal antibody preparation

Specific primers (S1 Table) were used to amplify the partial coding regions of *LmAbd1* and *LmAbd6*, which were subsequently subcloned into pET32a vector (Novagen, Germany). The recombinant pET32a vector was transformed into *Escherichia coli* BL21 (DE3) cells (TransGen, Beijing, China) and induced with IPTG. The target proteins were purified by affinity chromatography using Ni-NTA resin [54], and the purified target proteins were detected by 12% SDS-PAGE with concentration determination by the BCA method. Rabbits and mice were immunized with purified antigens to obtain rabbit polyclonal antibodies (anti-LmAbd-1 antibodies) and mouse polyclonal antibodies (anti-LmAbd-6 antibodies), respectively.

### Immunohistochemistry

Immunohistochemical staining was performed as described by Liu and colleagues [51]. Briefly, paraffin sections (5 μm) of the abdominal cuticle (including the ISM) of adult female locusts were prepared. The LmAbd-1 and LmAbd-6 proteins were detected in paraffin sections by incubation with the LmAbd-1 rabbit antiserum (1:200) and LmAbd-6 mouse antiserum (1:200) as a primary antibody at 4 °C overnight followed by washing with PBS three times for 5 min. The tissue samples were incubated with a diluent corresponding to the primary antibody and secondary antibody (antibody diluent was 1×PBS containing 1% BSA) 37 °C for more than 2 h. After washing the tissues three times with PBS, the specimens were incubated with Fluorescent Brightener 28 (FB28) (Sigma, USA) (1 mg/mL) for 5 s to detect chitin [55]. SYTOXR Green nucleic acid stain (Life Technologies, USA) was added to label the nuclei at a dilution of 1:50,000. The stained tissues were imaged using an LSM 880 confocal laser-scanning microscope (Zeiss, Oberkochen, Germany) at 60× magnification. All images from each staining experiment were collected under the same conditions.

### Measurement of extensibility

The abdomen of adult female or male locusts at day 10 after emergence was stretched by hand until the ISMs showed signs of breaking down, as previously described [14]. The mechanical properties of abdominal ISMs, including tensile strength (Maximum breaking force of ISM under tensile conditions, gf), maximum breaking strength (Maximum breaking force per unit of cross-sectional area, gf/mm^2^), and elongation at breaking (Tensile elongation of ISM at break, mm), were measured on day 10 post-emergence by a texture analyzer (TA.TOUCH, Baoseng, Shanghai, China).

### Juvenile hormone (JH) treatment

A JH analog, s-(+)-methoprene (50 μg/μL dissolved in acetone, Santa Cruz Biotech), was topically applied to adult females or males on day 2 post-emergence for 12 and 24 h, respectively. Acetone-only application served as a negative control for JH treatment. The abdominal ISMs from JH treatment and control groups were dissected for total RNA extraction and cDNA synthesis. All samples were collected with four biological replicates and each with four locusts. Expression of *LmAbd-1* and *LmAbd-6* was analyzed post-treatment by RT-qPCR as described above.

### Behavioral assay

The oviposition behavior of locusts in the control group and the treatment group was observed through video recording. The frequency of locust penetration into the soil, the time of egg laying, and the depth of egg laying under the soil were measured. The number of eggs (including oocysts and oocytes) was counted and number of residual oocytes in the abdomen was subsequently analyzed by dissecting female insects.

### Homology modeling and molecular docking

The Protein Data Bank (PDB) was initially searched to identify the best JHBP template for modeling the LmJHBP structure. BmJHBP (PDB ID: 2RQF.1A) from the silkworm *B. mori*, which can bind to JHIII, was chosen as an appropriate template for homology modeling of the LmJHBP structure using the Modeller 10.1 package (http://salilab.org/modeller/) [56]. Molecular docking was performed using the AutoDock Vina 1.1.2 program to determine the binding modes between LmJHBP and JHIII [57]. The PyMOL Molecular Graphics System version 2.2.0 (Schrödinger, LLC) was used for 3D visual analysis and to generate the LmJHBP-JHIII complexes.

### Statistical analysis

GraphPad 8 software (https://www.graphpad.com) was used for data analysis and graphing. Data statistics were analyzed using an independent sample Student *t* test (two-tailed unpaired); asterisks indicate significant differences (*p < 0.05, **p < 0.01, ***p < 0.001, ****p < 0.0001). The error bars in the figures represent the mean SEM for at least three separate biological experiments. Analysis of variance (ANOVA) was used to compare multiple sets of data; significant differences are represented by different lowercase letters (*p* < 0.05), and the specific analysis method is shown in the figure legends.

## Supporting information

S1 Fig The oviposition behavior of adult female locusts relies on extensible intersegmental membranes. **(a)** Schematic diagram of adult female locust oviposition behavior dependent on intersegmental membrane (ISM). **(b–d)** The locust abdomen extends long into the soil during oviposition. **(e, f)** The locust abdomen gradually shrinks to its normal level after laying eggs.
(TIF)

S2 Fig The microstructure difference of intersegment membranes between adult female and male locusts. Seg, Segment; ISM, Intersegmental membrane; FISM, female intersegmental membrane; MISM, male intersegmental membrane.
(TIF)

S3 Fig The structure of ISM4 and ISM6 in adult female locust. **(a, a′)** The epicuticle structural ISM4 and ISM6 was observed by SEM. **(b, b′)** The ultrastructural of ISM4 and ISM6 was observed by TEM. Epi, Epicuticle; el, Elastomer layer.
(TIF)

S4 Fig The structure of the segment in adult locust. **(a)** Schematic diagram of the structure of segment (Seg) modified according to Zhao and colleagues [23]. **(b)** The surface structure of Seg was observed by SEM. **(c)** The ultrastructure of Seg was observed by TEM. **(d)** The epidermal cells of Seg were observed by TEM. Seg, Segment; PC, Pore canal; Epi, Epicuticle; Exo, Exocuticle; Endo, Endocuticle; Ec, Epidermal cells; Nu, Nucleus.
(TIF)

S5 Fig Differentially expressed genes between segmental and intersegmental membranes based on transcriptome analysis. **(a)** Volcano map of differentially expressed genes (DEGs) between Seg and ISM. **(b)** Volcano map of DEGs involved in energy production, cuticle structure protein, chitin synthesis, lipid synthesis and transport, and cuticle tanning. **(c–f)** Heat map representing the gene-expression levels is involved in cuticle structure protein, chitin synthesis, lipid synthesis and transport, and cuticle tanning. Heat-map signal indicates log2 fold-change value relative to the mean expression level within the group. The red signal represents a higher expression, whereas the blue signal represents a lower expression. Seg and ISM represent segment and intersegmental membrane, respectively. The data underlying the graphs shown in the figure can be found in S2 Data.
(TIF)

S6 Fig Metabolomics analysis of differential metabolites between segmental and intersegmental membranes. **(a, a′)** Orthogonal projections to latent structures- discriminant analysis of differentially grouped between Seg and ISM in both positive and negative ion modes, respectively. **(b, b′)** Cluster heat map analysis of differential metabolites between Seg and ISM in both positive and negative ion modes, respectively. **(c, c′)** Volcano map of differential metabolites between Seg and ISM in both positive and negative ion modes, respectively. Seg and ISM represent segment and intersegmental membrane, respectively. The data underlying the graphs shown in the figure can be found in S3 Data.
(TIF)

S7 Fig The pathway of chitin synthase, cuticle tanning, and cuticle lipid synthase and transport. **(a)** The pathway of chitin synthase. **(b)** The cuticle tanning pathway. **(c)** The cuticle lipid synthase and transport pathway. **(d)** Schematic diagram of segment and intersegmental membrane structure modified according to Zhao and colleagues [23].
(TIF)

S8 Fig Analysis of *LmAbd-1* and *LmAbd-6* expression and sequence. **(a, a′)** The difference expression of *LmAbd-6* in adult female and male ISM based on transcriptome and RT-qPCR results. **(b, b′)** The expression of *LmAbd-1* in adult female and male ISM based on transcriptome and RT-qPCR results. *n* = 3 biological replicates. **(c)** The expression of *LmAbd-1* and *LmAbd-6* in different female ISMs by RT-qPCR. *n* = 3 biological replicates. Different lowercase letters above the error bars (a, b, c, and d) represent significant differences by one-way ANOVA (Tukey HSD multiple comparisons test, *P* < 0.05). The data underlying the graphs shown in the figure can be found in S2 Data. **(d)** Gene sequence analysis of *LmAbd-1* and *LmAbd-6*. **(e)** Amino acid sequence analysis of LmAbd-1 and LmAbd-6. The red box showed the signal peptide (SP), and the green box showed the chitin-binding domain 4 (ChtBD4).
(TIF)

S9 Fig Homology analysis of Abd-1 and Abd-6 in different species. **(a)** Multiple sequence alignments of LmAbd-1 and Abd-1 from different insect species. **(b)** Multiple sequence alignments of LmAbd-6 and Abd-6 from different insect species. The orange box showed the signal peptide (SP), and the green box showed the RR-1 motif. Lm, *Locusta migratoria*; Sg, *Schistocerca gregaria*; Sa, *Schistocerca americana*; As, *Atractomorpha sinensis*; Cn, *Ceracris nigricornis*; Xb, *Xenocatantops brachycerus*; Ts, *Tetrix subulata*. **(c)** Analysis of RR-1 motifs using the Weblogo tool.
(TIF)

S10 Fig Phylogenetic tree of Abd-1 and Abd-6 in different species. (TIF)

S11 Fig The silencing efficiency of *LmAbd-1* and *LmAbd-6* and phenotype of adult female locusts. **(a, b)** The silencing efficiency of *LmAbd-1* and *LmAbd-6*. *n* = 4 biological replicates. Student *t* test (two-tailed) was applied for two-group comparisons. The data are shown as the mean ± SEM. The data underlying the graphs shown in the figure can be found in S2 Data. **(c)** The phenotypic of locusts in the adult stage after knockdown of *LmAbd-1* and *LmAbd-6*.
(TIF)

S12 Fig The structure of the fourth and sixth female intersegmental membranes. **(a)** The epicuticle structure of the fourth and sixth female ISMs was observed by SEM after knockdown of *LmAbd-1* and *LmAbd-6*. **(b)** The ultrastructural of the fourth and sixth female ISMs were observed by TEM after knockdown of *LmAbd-1* and *LmAbd-6*. epi: epicuticle; el: elastomer layer.
(TIF)

S13 Fig The number of eggs and the size of the remaining oocysts in adult female locusts. **(a–a**″**)** The size of the remaining oocysts in adult female locusts after injection of ds*LmAbd-1* and ds*LmAbd-6* compared to that of control. **(b–b**″**)** The number of eggs after injection of ds*LmAbd-1* and ds*LmAbd-6* compared to that of control.
(TIF)

S14 Fig The expression level of *LmKr-h1* and tensile strength of adult female ISM5. **(a)** The expression of *LmKr-h1* in different female ISMs by RT-qPCR. *n* = 3 biological replicates. Different lowercase letters above the error bars (a and b) represent significant differences by one-way ANOVA (Tukey HSD multiple comparisons test, *P* < 0.05). **(b)** The microstructure difference of female intersegment membrane 5 (ISM5) between *LmKr-h1* RNAi treatment and control groups. **(c–c**″**)** Tensile strength, breaking strength, and elongation at break of female ISM5 after knockdown of *LmKr-h1*. *n* = 19 biologically independent female locusts. Student *t* test (two-tailed) was applied for two-group comparisons. The data are shown as the mean ± SEM. The data underlying the graphs shown in the figure can be found in S2 Data.
(TIF)

S15 Fig The expression levels of *LmAbd-1* and *LmAbd-6* and the extensibility of adult male ISM5 were determined after induced by JHA in male locusts. **(a)** The expression of *LmAbd-1* and *LmAbd-6* were induced by JHA in adult males. *n* = 3 biological replicates. **(b, b′)** The microstructure difference (width) of male intersegment membrane 5 (ISM5) between JHA treatment and control groups. **(c–c**″**)** The tensile strength, breaking strength and elongation at break of male ISM5 after induction of JHA. *n* = 19 biologically independent locusts. Student *t* test (two-tailed) was applied for other two-group comparisons. The data are shown as the mean ± SEM. The data underlying the graphs shown in the figure can be found in S2 Data.
(TIF)

S16 Fig Expression and homology analysis of JHBP in different species. **(a, a′)** The difference expression of *LmJHBP* in adult female and male ISM based on transcriptome and RT-qPCR results. **(b)** The expression of *LmJHBP* in different female ISMs by RT-qPCR. *n* = 3 biological replicates. Different lowercase letters above the error bars (a, b, c, and d) represent significant differences by one-way ANOVA (Tukey HSD multiple comparisons test, *P* < 0.05). The data underlying the graphs shown in the figure can be found in S2 Data. **(c)** The phylogenetic tree of JHBP in different species. **(d)** Multiple sequence alignments of LmJHBP and JHBP from different insect species. The red box represents signal peptide (SP), and the orange boxes represent juvenile hormone binding protein (JHBP). Lm, *Locusta migratoria*; Sg, *Schistocerca gregaria*; Sa, *Schistocerca americana*; Sn, *Schistocerca nitens*; Sc, *Schistocerca cancellata*.
(TIF)

S17 Fig Homologous modeling of LmJHBP and BmJHBP. **(a)** Structure pattern diagram of LmJHBP protein. **(b)** Structure pattern diagram of LmJHBP+BmJHBP protein. **(c)** Structure pattern diagram of BmJHBP protein binding to JHIII. (d) Structure pattern diagram of LmJHBP protein binding to JHIII.
(TIF)

S18 Fig The expression level of *LmJHBP* and tensile strength of adult female ISM5. **(a, b)** The expression level of *LmJHBP* after injection of ds*GFP*, ds*LmMet*, and ds*LmKr-h1.* **(c, c′)** Tensile strength of female ISM5 after knockdown of *LmJHBP*. *n* ≥ 17 biologically independent locusts. **(d)** Breaking strength of female ISM5 after knockdown of *LmJHBP*. *n* ≥ 17 biologically independent locusts. Student *t* test (two-tailed) was applied for two-group comparisons. The data are shown as the mean ± SEM. ns, no significant difference. The data underlying the graphs shown in the figure can be found in S2 Data.
(TIF)

S19 Fig The size of the remaining oocysts in adult female locusts after knockdown of *LmJHBP.* **(a, b)** The size of the remaining oocysts in adult female locusts after injection of ds*LmJHBP* compared to that of control.
(TIF)

S20 Fig Microstructure difference of intersegment membranes (ISM 4 and ISM 6) after knockdown of *Tra-2* or *Dsx.* **(a)** The microstructure difference of female ISM4 and ISM6 between *Tra-2* or *Dsx* RNAi treatment and control groups. **(b)** The microstructure difference of male ISM4 and ISM6 between *Dsx* RNAi treatment and control groups.
(TIF)

S1 Video The oviposition behavior of mature adult female locust, related to S1A Fig. (MP4)

S2 Video The extensibility of ISM5 between adult female and male locust, related to Fig 1D. (MP4)

S3 Video The extensibility of adult female ISM5 after knockdown of *LmAbd-1*, related to Fig 4B and 4C. (MP4)

S4 Video The extensibility of adult female ISM5 after knockdown of *LmAbd-6*, related to Fig 4B and 4C. (MP4)

S5 Video The extensibility of adult female ISM5 after knockdown of *LmJHBP*, related to Fig 5L. (MP4)

S1 Table Primer sequences used in this study. (DOCX)

S2 Table Species and GenBank accession number for phylogenetic tree used in this study. (DOCX)

S1 Data Related to Figs 1–6. (XLSX)

S2 Data Related to S5 and S8–S18 Figs. (XLSX)

S3 Data Related to S6 Fig. (XLSX)

## Abbreviations

ANOVA: analysis of variance
CPs: cuticle proteins
DEGs: differentially expressed genes
GO: Gene Ontology
ISMs: intersegmental membranes
JH: juvenile hormone
LC-MS: liquid chromatography–mass spectrometry
OPLS-DA: orthogonal projections to latent structures-discriminant analysis
PCA: principal component analysis
RT-qPCR: real-time quantitative PCR
SEM: scanning electron microscopy
TEM: transmission electron microscopy
UPLC-MS: ultrahigh-performance liquid chromatography-high-resolution mass spectrometry
