# Supplementary figures and images for: The Tra/Dsx-JHBP axis controls female-specific gene expression and oviposition in locusts
Source: PLoS Biol. 2025 Aug 5;23(8):e3003321. doi: 10.1371/journal.pbio.3003321 (PMC12349703; doi:10.1371/journal.pbio.3003321)

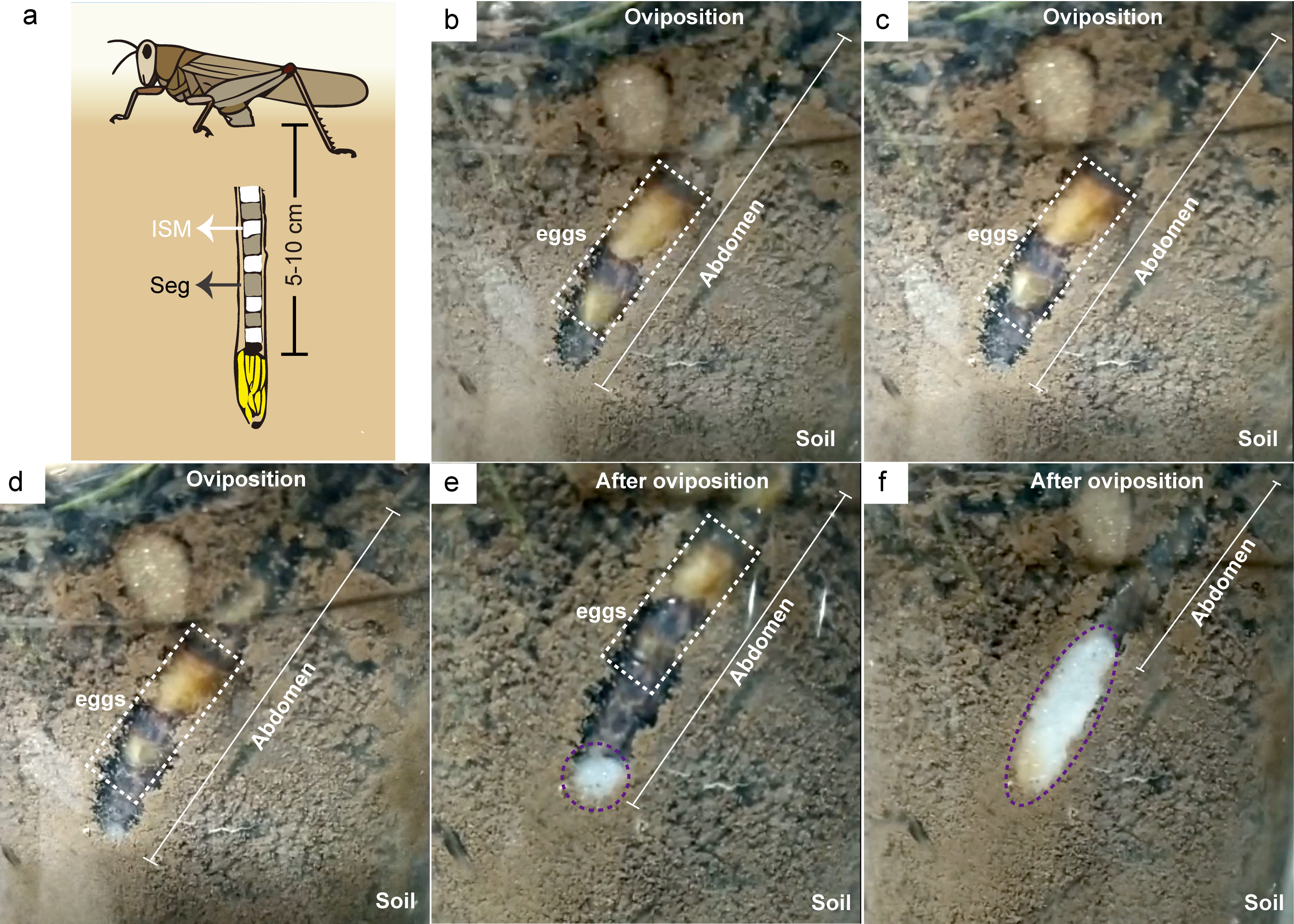

Supplement: S1 Fig — (a) Schematic diagram of adult female locust oviposition behavior dependent on intersegmental membrane (ISM). (b–d) The locust abdomen extends long into the soil during oviposition. (e, f) The locust abdomen gradually shrinks to its normal level after laying eggs. (TIF) [file pbio.3003321.s001.tif]

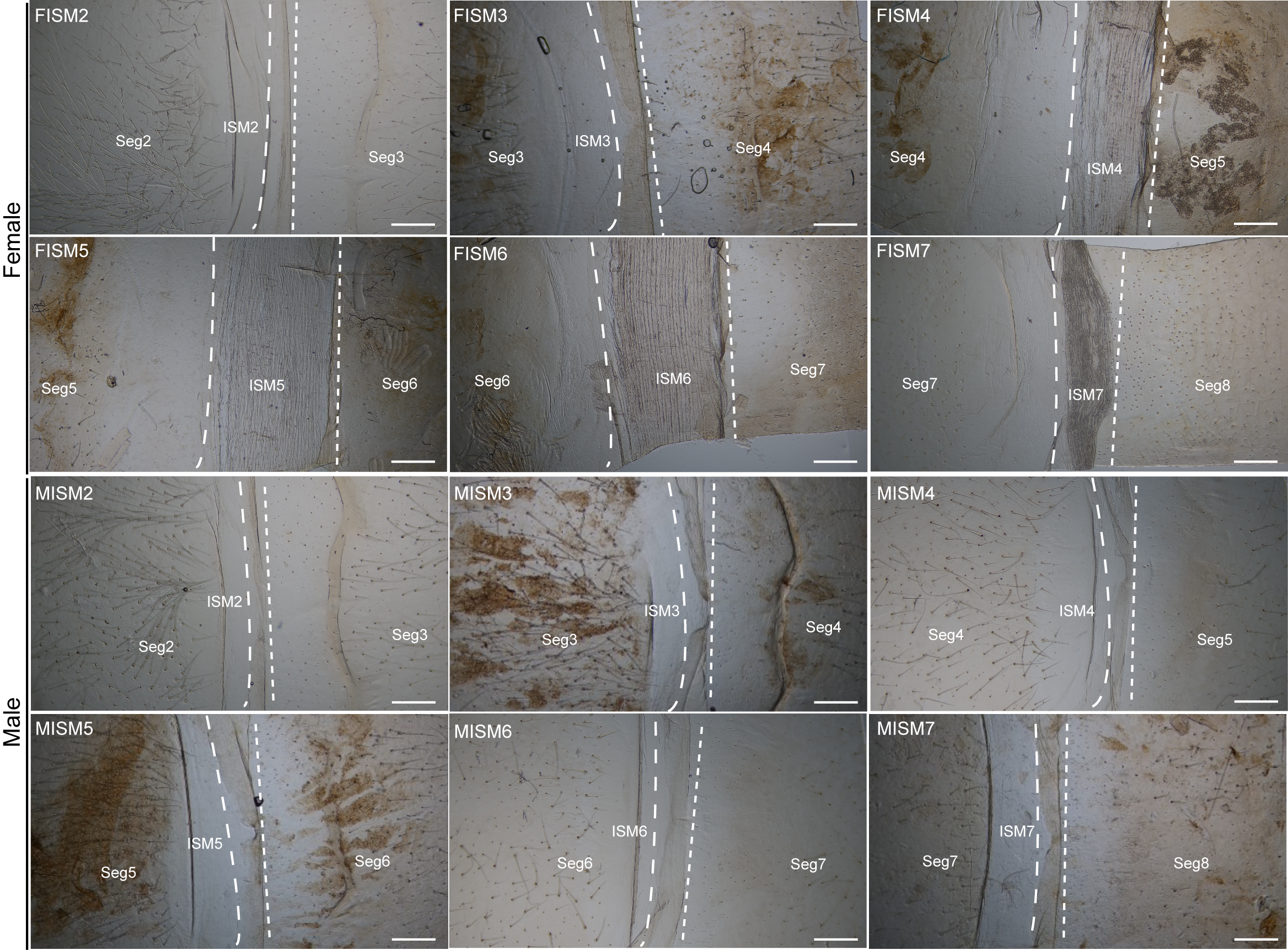

Supplement: S2 Fig — Seg, Segment; ISM, Intersegmental membrane; FISM, female intersegmental membrane; MISM, male intersegmental membrane. (TIF) [file pbio.3003321.s002.tif]

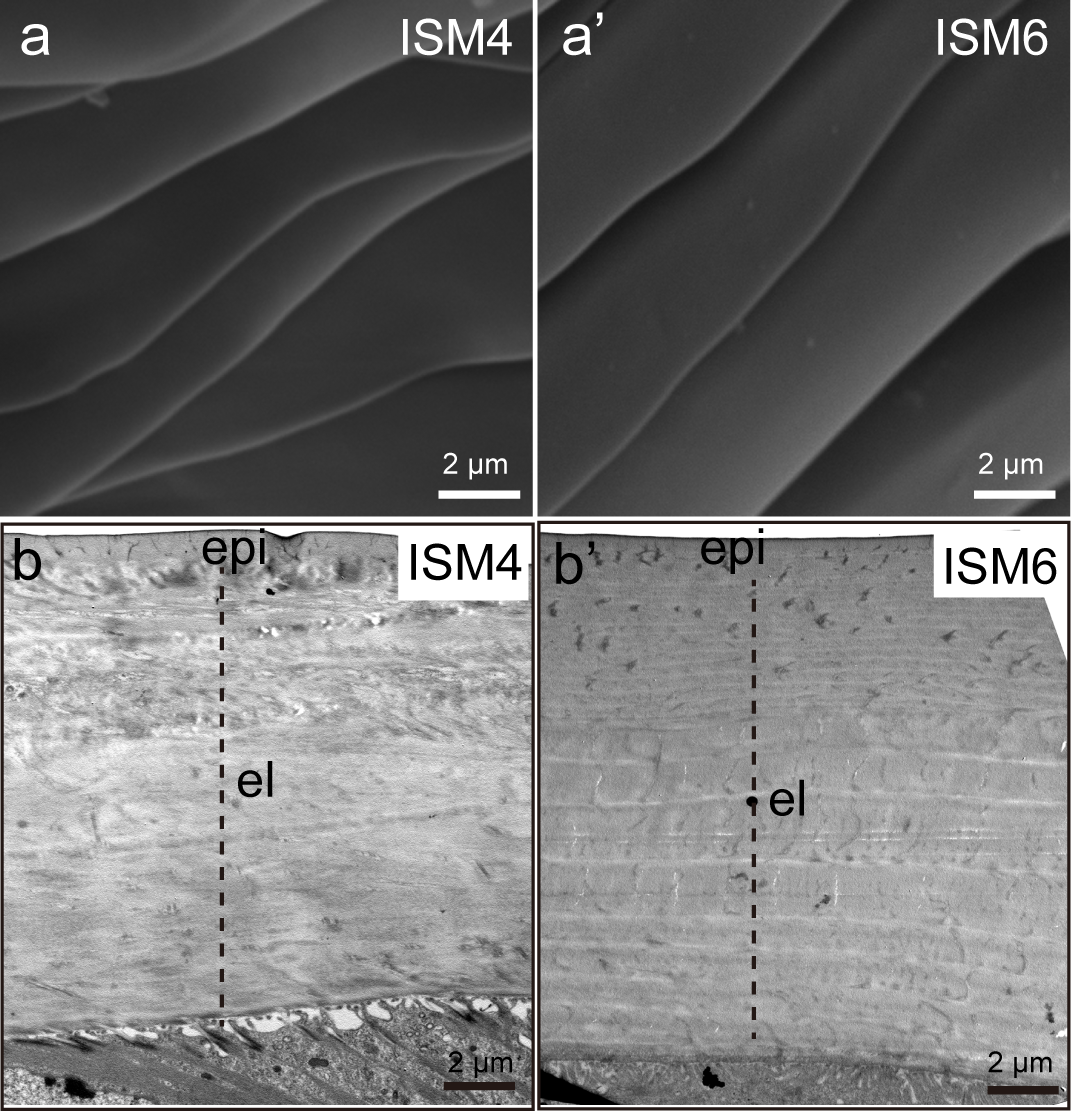

Supplement: S3 Fig — (a, a′) The epicuticle structural ISM4 and ISM6 was observed by SEM. (b, b′) The ultrastructural of ISM4 and ISM6 was observed by TEM. Epi, Epicuticle; el, Elastomer layer. (TIF) [file pbio.3003321.s003.tif]

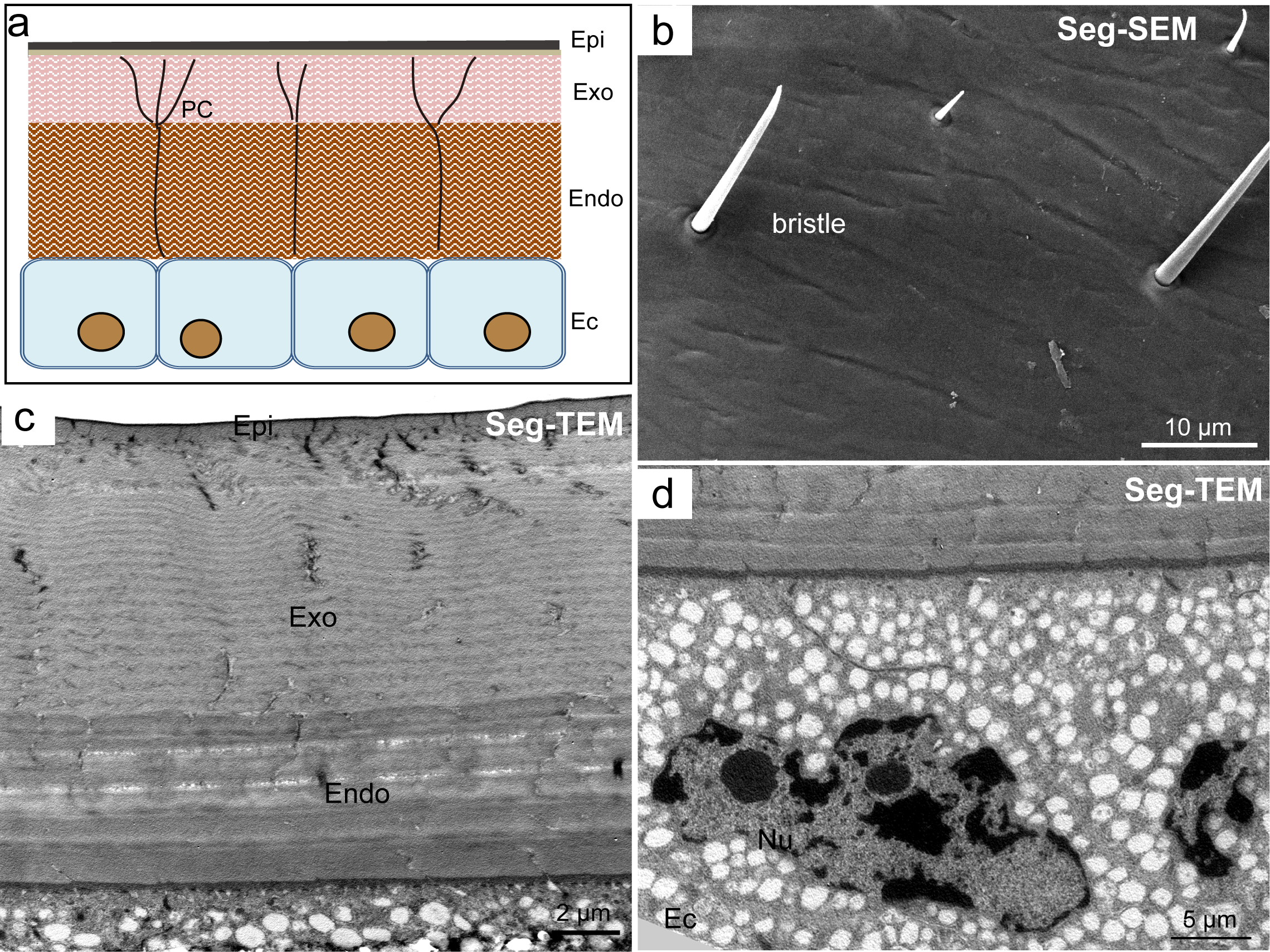

Supplement: S4 Fig — (a) Schematic diagram of the structure of segment (Seg) modified according to Zhao and colleagues [23]. (b) The surface structure of Seg was observed by SEM. (c) The ultrastructure of Seg was observed by TEM. (d) The epidermal cells of Seg were observed by TEM. Seg, Segment; PC, Pore canal; Epi, Epicuticle; Exo, Exocuticle; Endo, Endocuticle; Ec, Epidermal cells; Nu, Nucleus. (TIF) [file pbio.3003321.s004.tif]

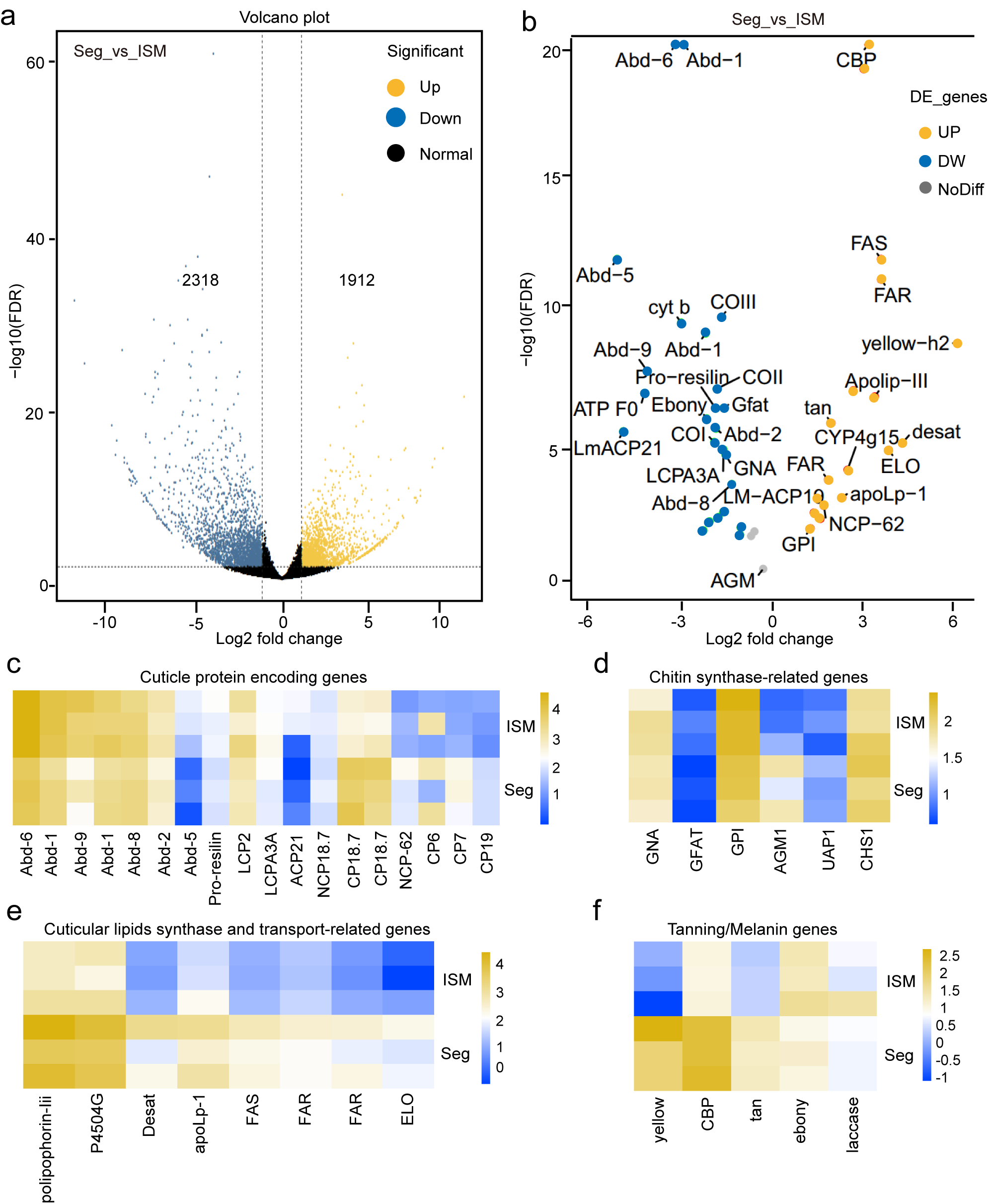

Supplement: S5 Fig — (a) Volcano map of differentially expressed genes (DEGs) between Seg and ISM. (b) Volcano map of DEGs involved in energy production, cuticle structure protein, chitin synthesis, lipid synthesis and transport, and cuticle tanning. (c–f) Heat map representing the gene-expression levels is involved in cuticle structure protein, chitin synthesis, lipid synthesis and transport, and cuticle tanning. Heat-map signal indicates log2 fold-change value relative to the mean expression level within the group. The red signal represents a higher expression, whereas the blue signal represents a lower expression. Seg and ISM represent segment and intersegmental membrane, respectively. The data underlying the graphs shown in the figure can be found in S2 Data. (TIF) [file pbio.3003321.s005.tif]

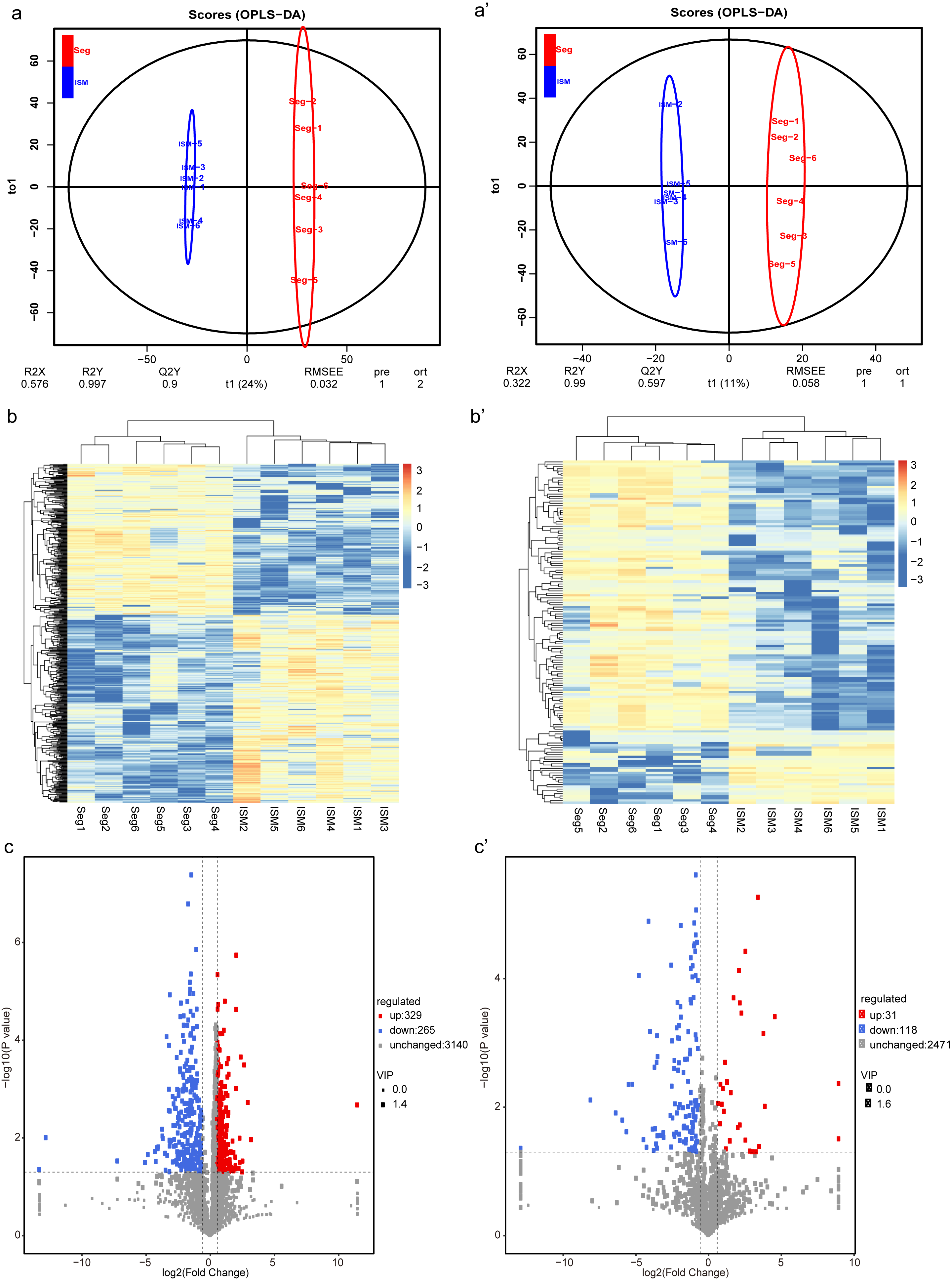

Supplement: S6 Fig — (a, a′) Orthogonal projections to latent structures- discriminant analysis of differentially grouped between Seg and ISM in both positive and negative ion modes, respectively. (b, b′) Cluster heat map analysis of differential metabolites between Seg and ISM in both positive and negative ion modes, respectively. (c, c′) Volcano map of differential metabolites between Seg and ISM in both positive and negative ion modes, respectively. Seg and ISM represent segment and intersegmental membrane, respectively. The data underlying the graphs shown in the figure can be found in S3 Data. (TIF) [file pbio.3003321.s006.tif]

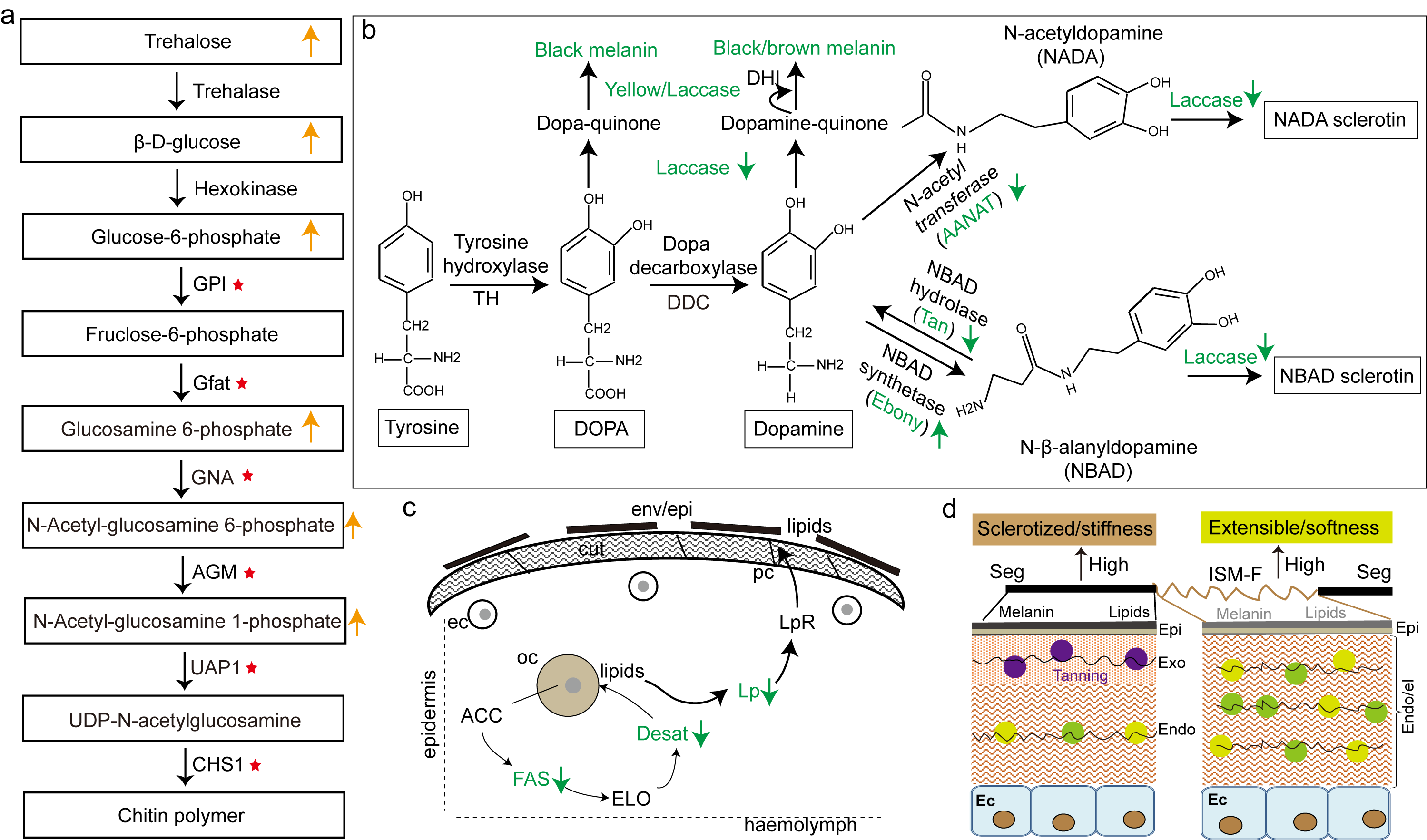

Supplement: S7 Fig — (a) The pathway of chitin synthase. (b) The cuticle tanning pathway. (c) The cuticle lipid synthase and transport pathway. (d) Schematic diagram of segment and intersegmental membrane structure modified according to Zhao and colleagues [23]. (TIF) [file pbio.3003321.s007.tif]

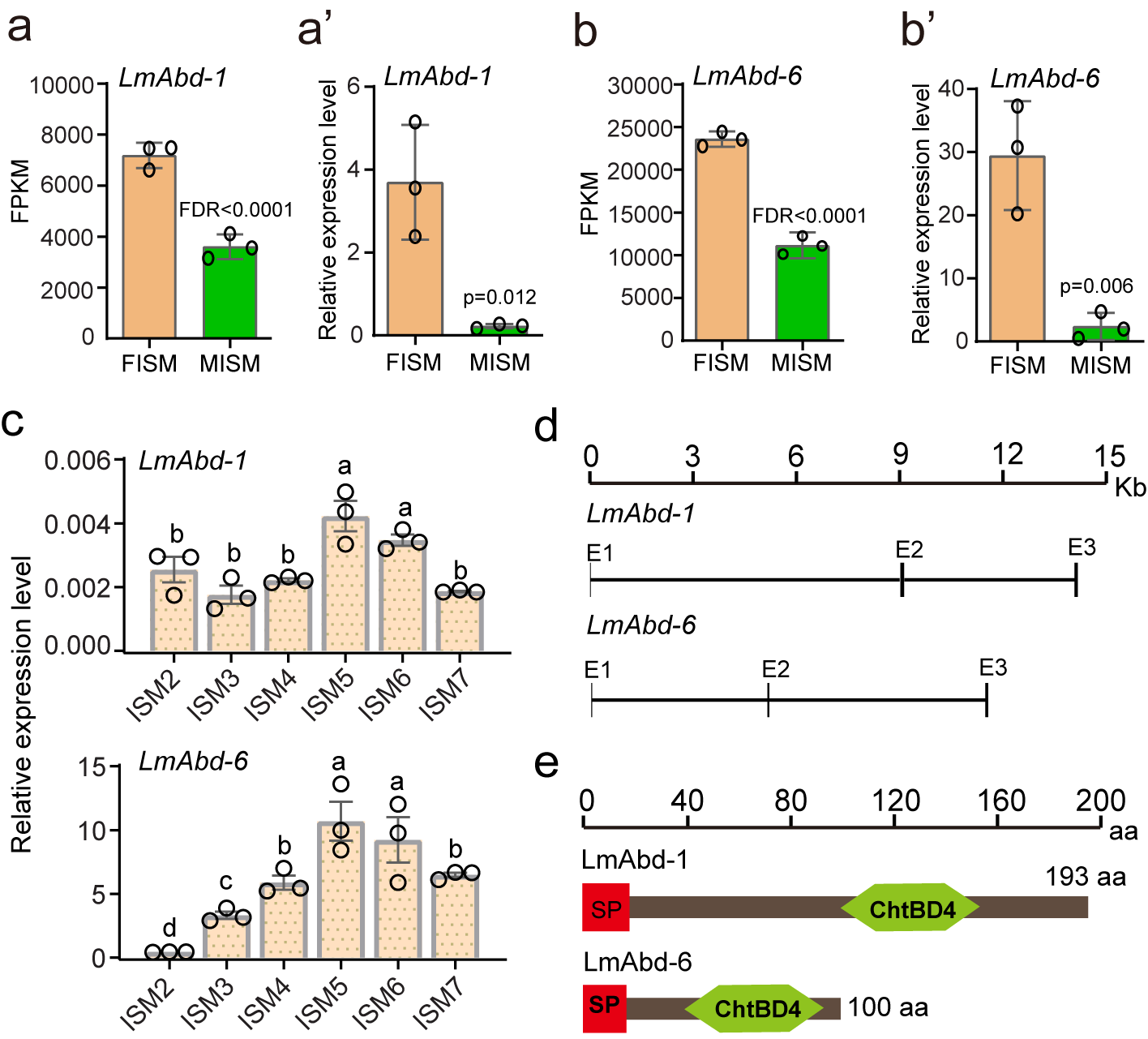

Supplement: S8 Fig — (a, a′) The difference expression of LmAbd-6 in adult female and male ISM based on transcriptome and RT-qPCR results. (b, b′) The expression of LmAbd-1 in adult female and male ISM based on transcriptome and RT-qPCR results. n = 3 biological replicates. (c) The expression of LmAbd-1 and LmAbd-6 in different female ISMs by RT-qPCR. n = 3 biological replicates. Different lowercase letters above the error bars (a, b, c, and d) represent significant differences by one-way ANOVA (Tukey HSD multiple comparisons test, P < 0.05). The data underlying the graphs shown in the figure can be found in S2 Data. (d) Gene sequence analysis of LmAbd-1 and LmAbd-6. (e) Amino acid sequence analysis of LmAbd-1 and LmAbd-6. The red box showed the signal peptide (SP), and the green box showed the chitin-binding domain 4 (ChtBD4). (TIF) [file pbio.3003321.s008.tif]

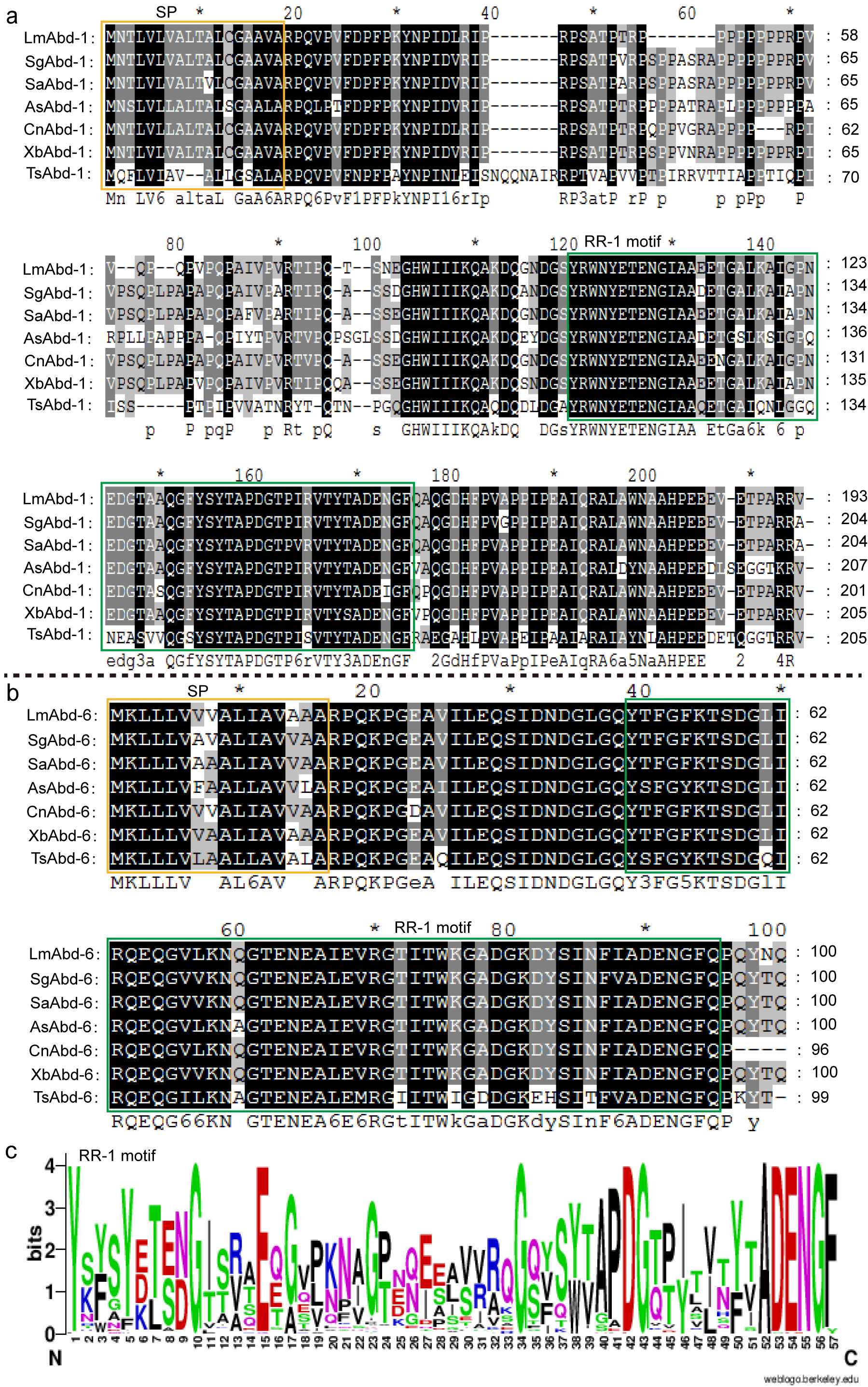

Supplement: S9 Fig — (a) Multiple sequence alignments of LmAbd-1 and Abd-1 from different insect species. (b) Multiple sequence alignments of LmAbd-6 and Abd-6 from different insect species. The orange box showed the signal peptide (SP), and the green box showed the RR-1 motif. Lm, Locusta migratoria; Sg, Schistocerca gregaria; Sa, Schistocerca americana; As, Atractomorpha sinensis; Cn, Ceracris nigricornis; Xb, Xenocatantops brachycerus; Ts, Tetrix subulata. (c) Analysis of RR-1 motifs using the Weblogo tool. (TIF) [file pbio.3003321.s009.tif]

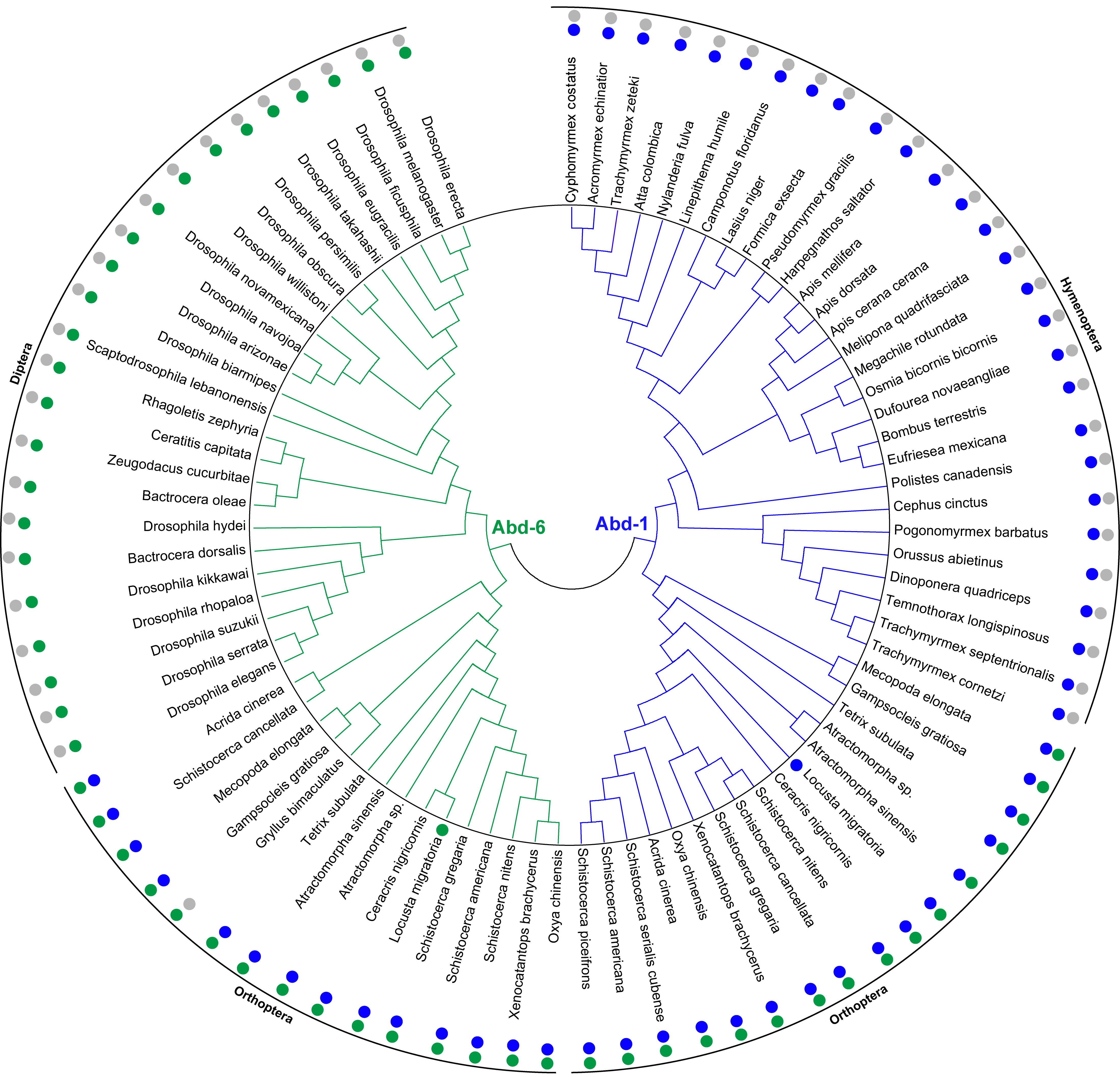

Supplement: S10 Fig — (TIF) [file pbio.3003321.s010.tif]

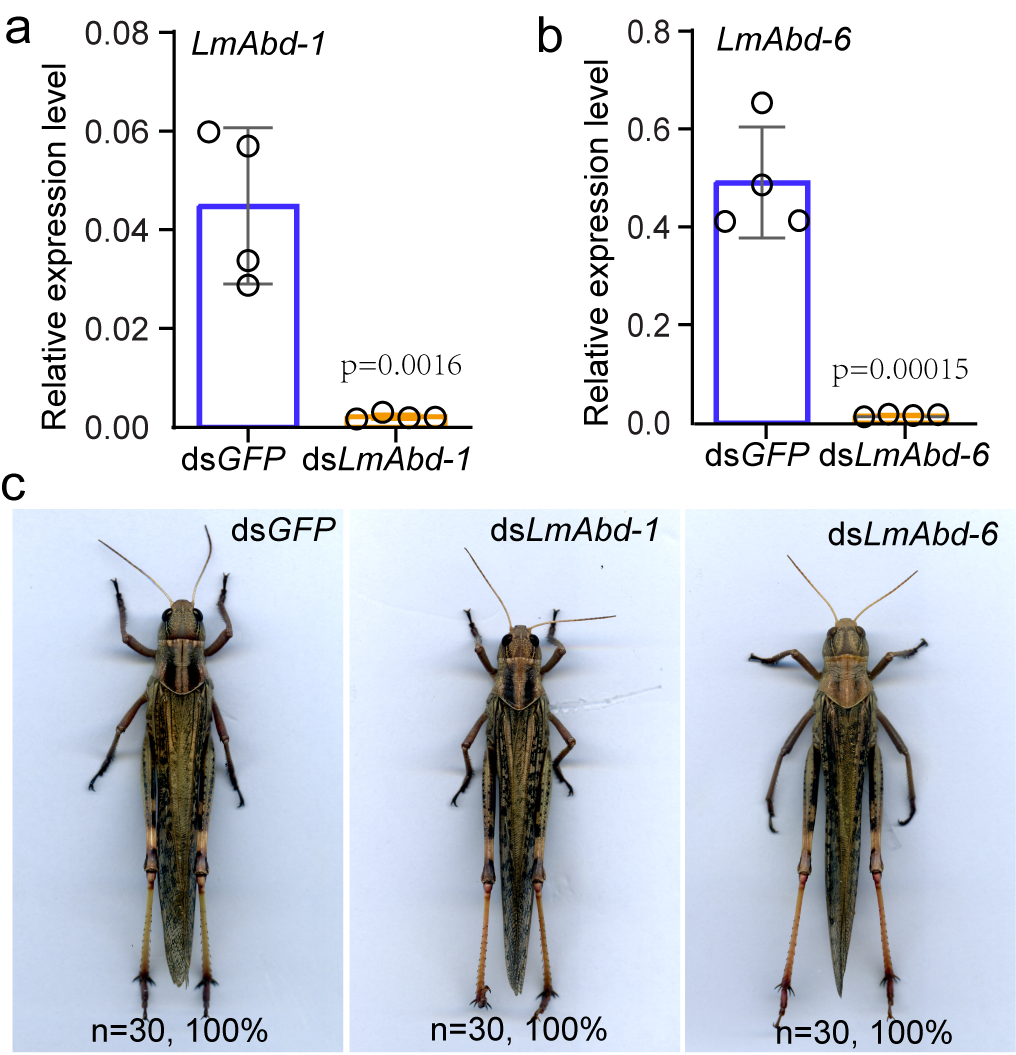

Supplement: S11 Fig — (a, b) The silencing efficiency of LmAbd-1 and LmAbd-6. n = 4 biological replicates. Student t test (two-tailed) was applied for two-group comparisons. The data are shown as the mean ± SEM. The data underlying the graphs shown in the figure can be found in S2 Data. (c) The phenotypic of locusts in the adult stage after knockdown of LmAbd-1 and LmAbd-6. (TIF) [file pbio.3003321.s011.tif]

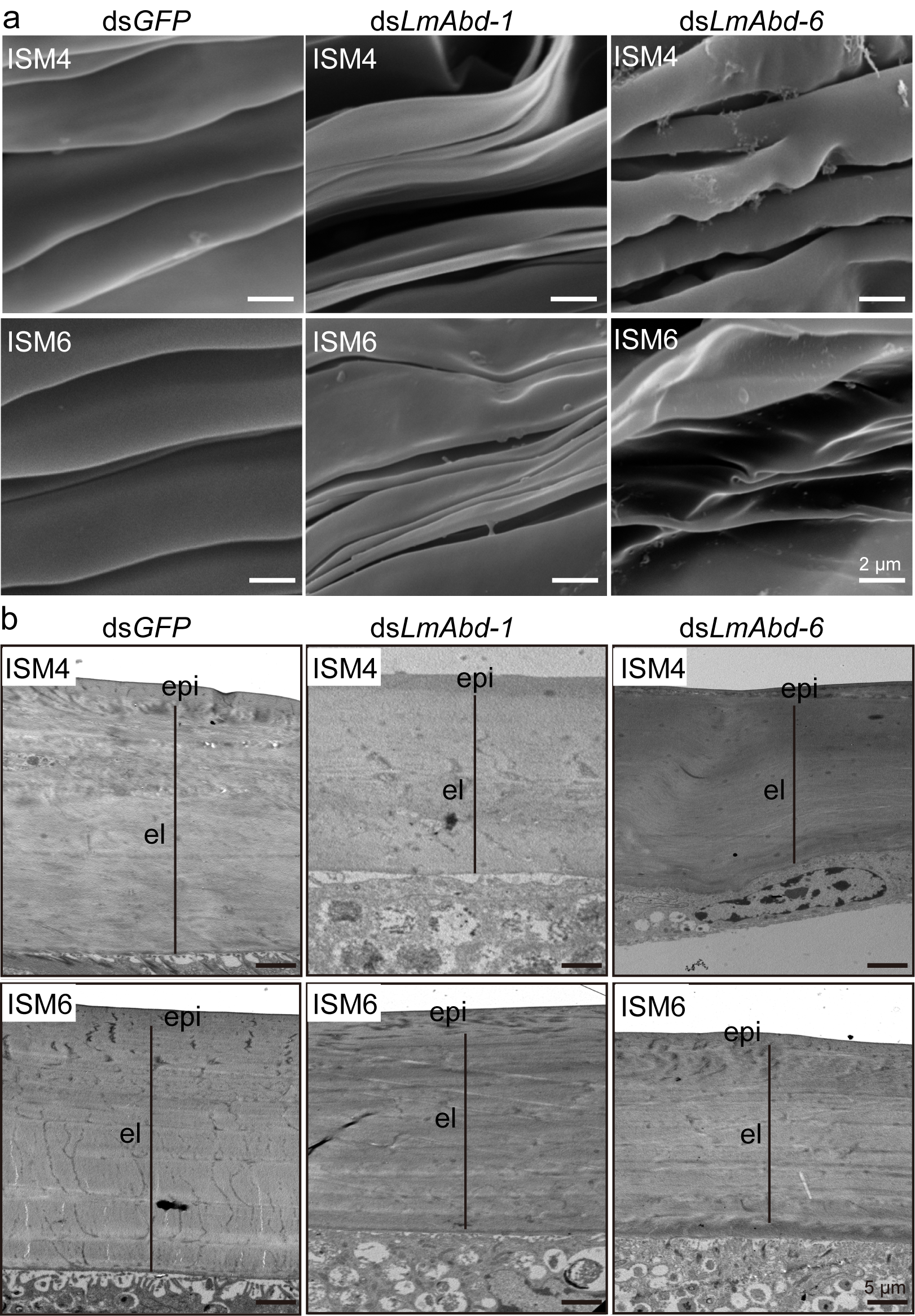

Supplement: S12 Fig — (a) The epicuticle structure of the fourth and sixth female ISMs was observed by SEM after knockdown of LmAbd-1 and LmAbd-6. (b) The ultrastructural of the fourth and sixth female ISMs were observed by TEM after knockdown of LmAbd-1 and LmAbd-6. epi: epicuticle; el: elastomer layer. (TIF) [file pbio.3003321.s012.tif]

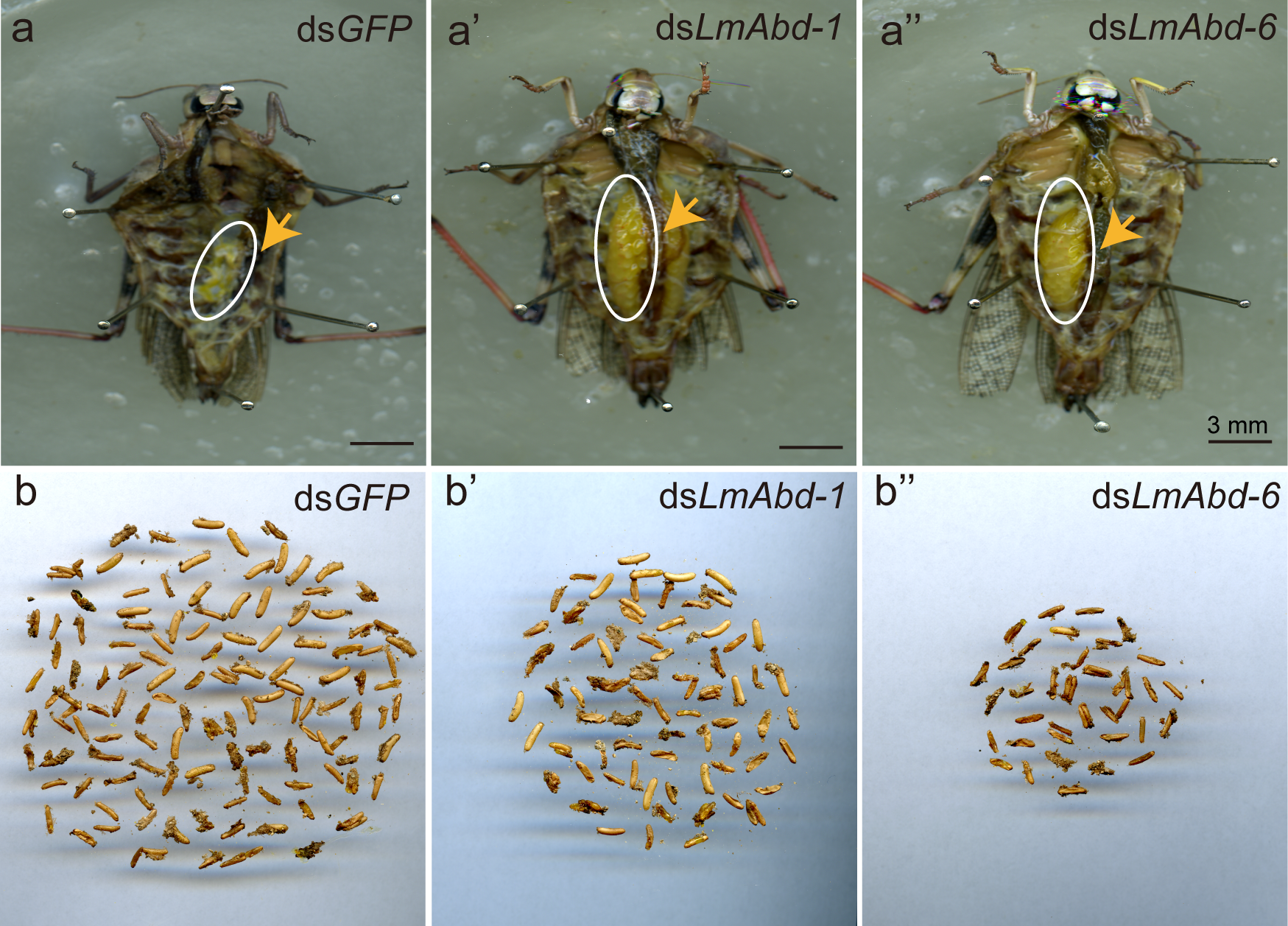

Supplement: S13 Fig — (a–a″) The size of the remaining oocysts in adult female locusts after injection of dsLmAbd-1 and dsLmAbd-6 compared to that of control. (b–b″) The number of eggs after injection of dsLmAbd-1 and dsLmAbd-6 compared to that of control. (TIF) [file pbio.3003321.s013.tif]

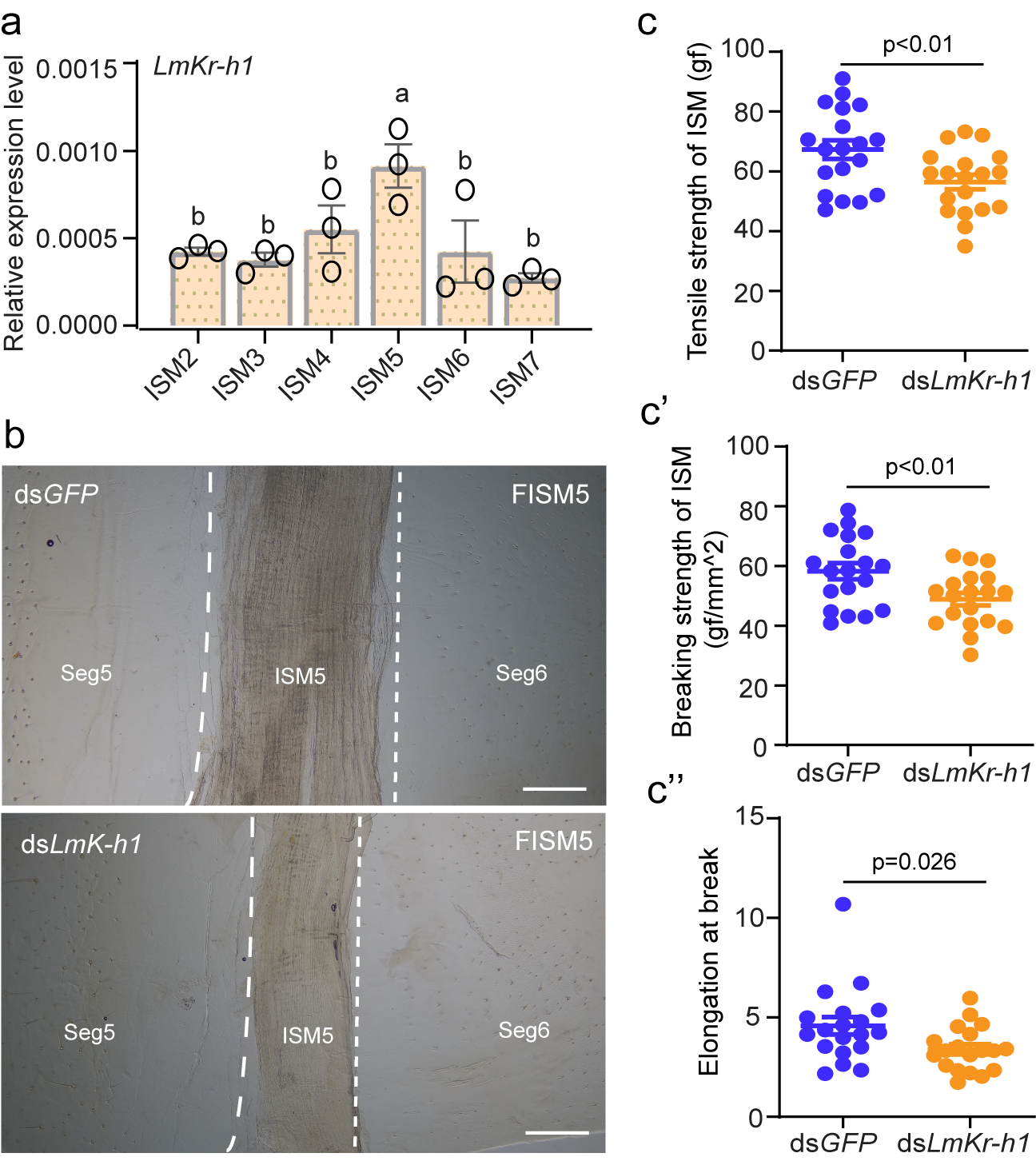

Supplement: S14 Fig — (a) The expression of LmKr-h1 in different female ISMs by RT-qPCR. n = 3 biological replicates. Different lowercase letters above the error bars (a and b) represent significant differences by one-way ANOVA (Tukey HSD multiple comparisons test, P < 0.05). (b) The microstructure difference of female intersegment membrane 5 (ISM5) between LmKr-h1 RNAi treatment and control groups. (c–c″) Tensile strength, breaking strength, and elongation at break of female ISM5 after knockdown of LmKr-h1. n = 19 biologically independent female locusts. Student t test (two-tailed) was applied for two-group comparisons. The data are shown as the mean ± SEM. The data underlying the graphs shown in the figure can be found in S2 Data. (TIF) [file pbio.3003321.s014.tif]

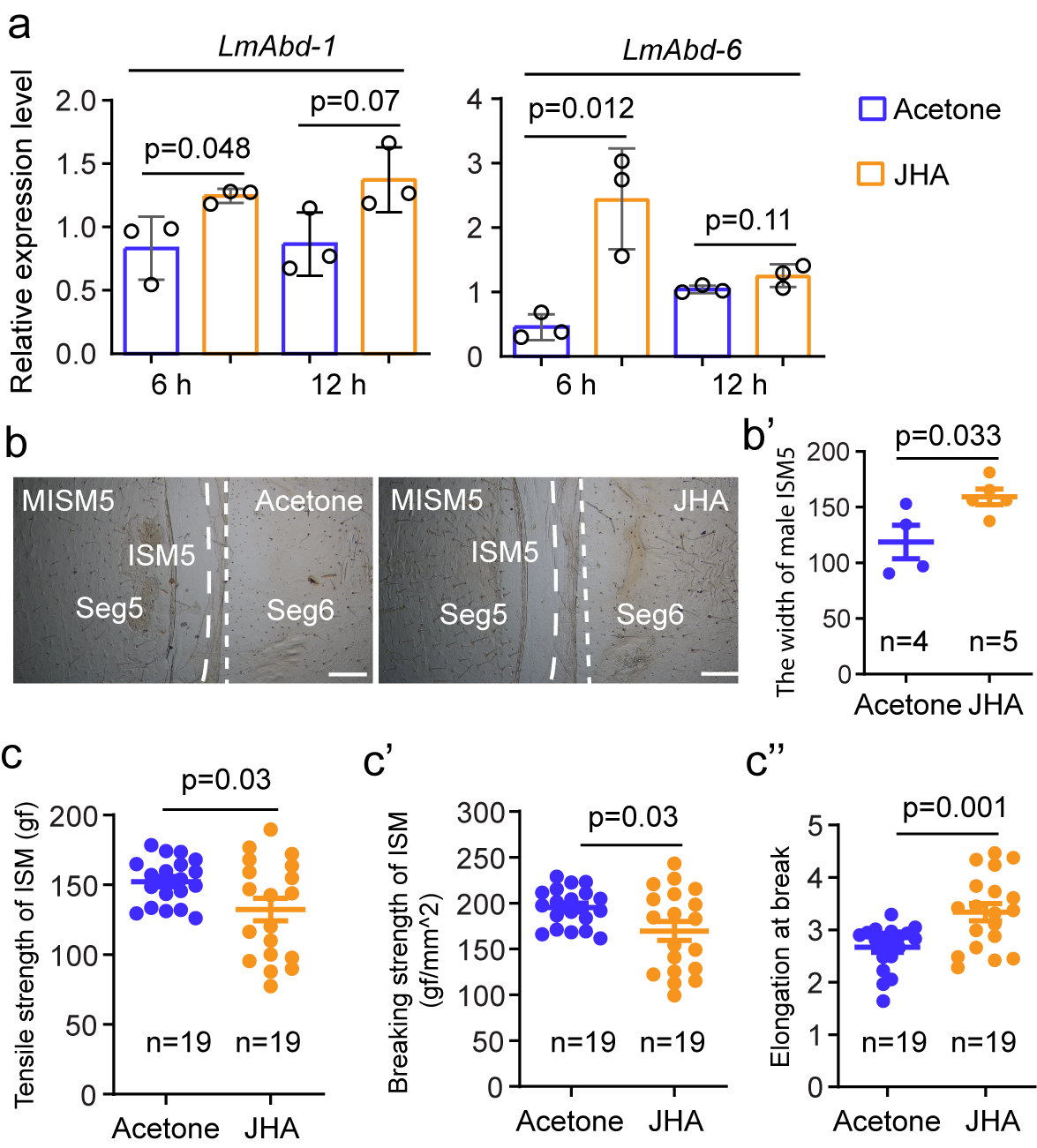

Supplement: S15 Fig — (a) The expression of LmAbd-1 and LmAbd-6 were induced by JHA in adult males. n = 3 biological replicates. (b, b′) The microstructure difference (width) of male intersegment membrane 5 (ISM5) between JHA treatment and control groups. (c–c″) The tensile strength, breaking strength and elongation at break of male ISM5 after induction of JHA. n = 19 biologically independent locusts. Student t test (two-tailed) was applied for other two-group comparisons. The data are shown as the mean ± SEM. The data underlying the graphs shown in the figure can be found in S2 Data. (TIF) [file pbio.3003321.s015.tif]

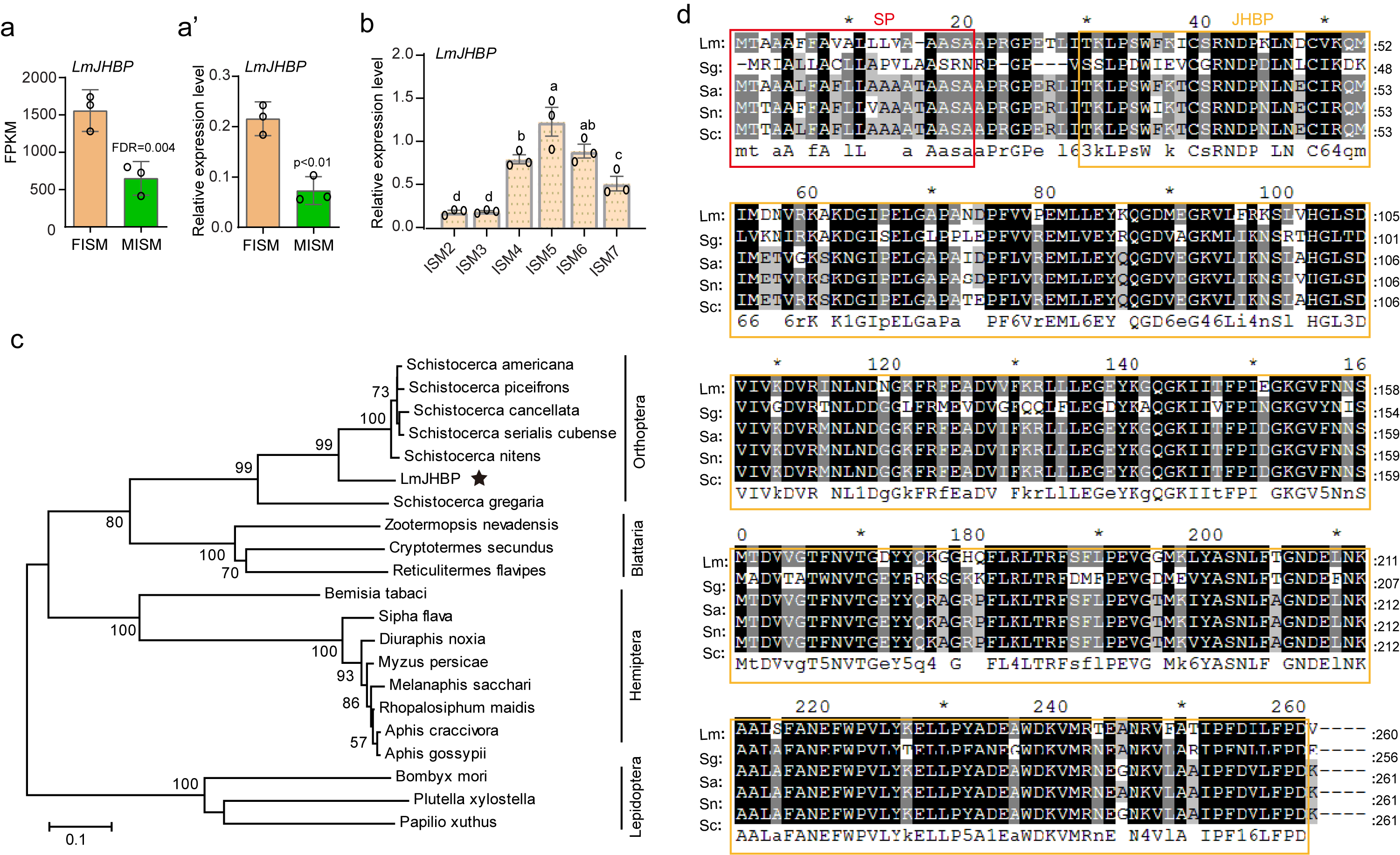

Supplement: S16 Fig — (a, a′) The difference expression of LmJHBP in adult female and male ISM based on transcriptome and RT-qPCR results. (b) The expression of LmJHBP in different female ISMs by RT-qPCR. n = 3 biological replicates. Different lowercase letters above the error bars (a, b, c, and d) represent significant differences by one-way ANOVA (Tukey HSD multiple comparisons test, P < 0.05). The data underlying the graphs shown in the figure can be found in S2 Data. (c) The phylogenetic tree of JHBP in different species. (d) Multiple sequence alignments of LmJHBP and JHBP from different insect species. The red box represents signal peptide (SP), and the orange boxes represent juvenile hormone binding protein (JHBP). Lm, Locusta migratoria; Sg, Schistocerca gregaria; Sa, Schistocerca americana; Sn, Schistocerca nitens; Sc, Schistocerca cancellata. (TIF) [file pbio.3003321.s016.tif]

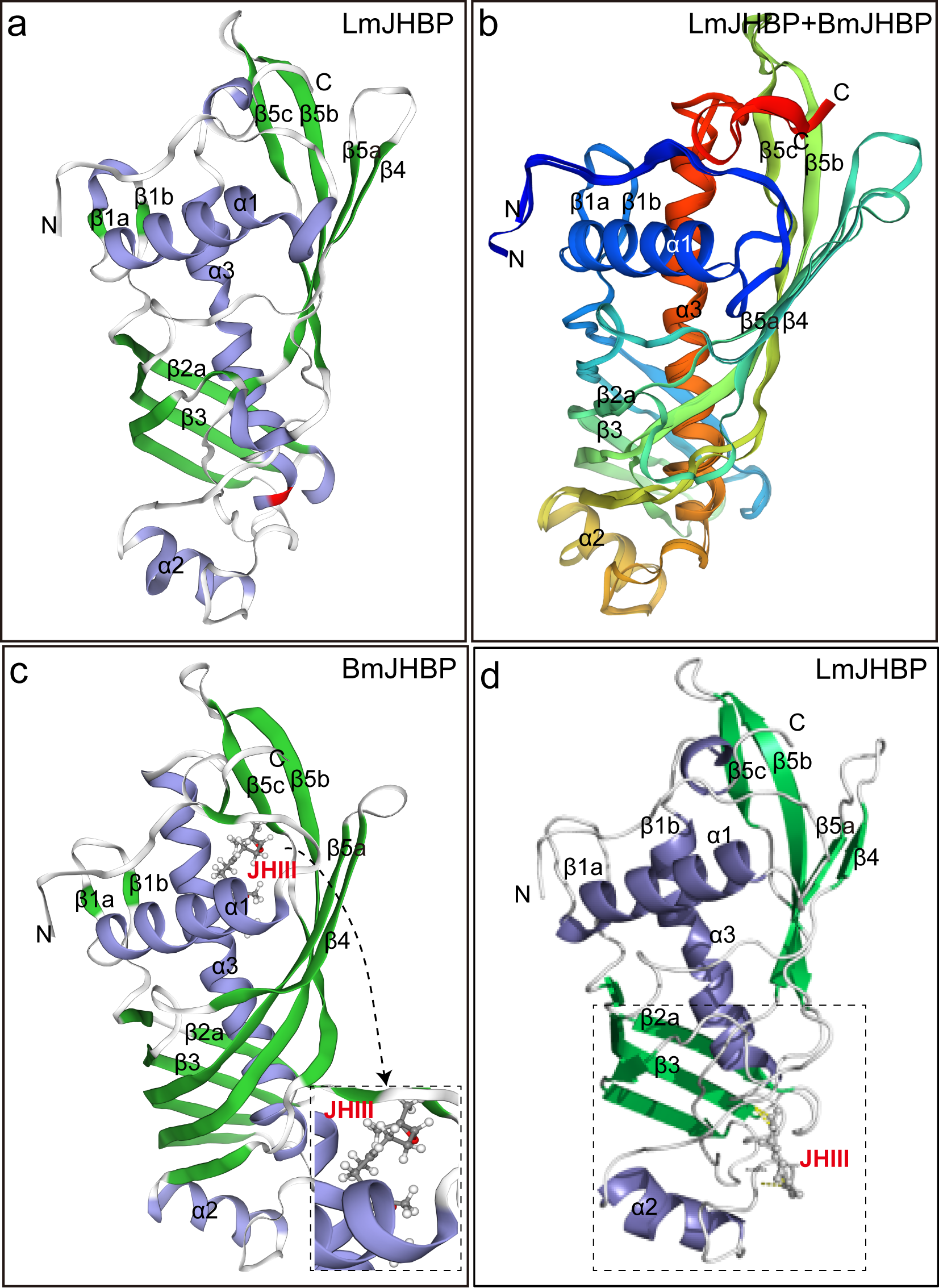

Supplement: S17 Fig — (a) Structure pattern diagram of LmJHBP protein. (b) Structure pattern diagram of LmJHBP+BmJHBP protein. (c) Structure pattern diagram of BmJHBP protein binding to JHIII. (d) Structure pattern diagram of LmJHBP protein binding to JHIII. (TIF) [file pbio.3003321.s017.tif]

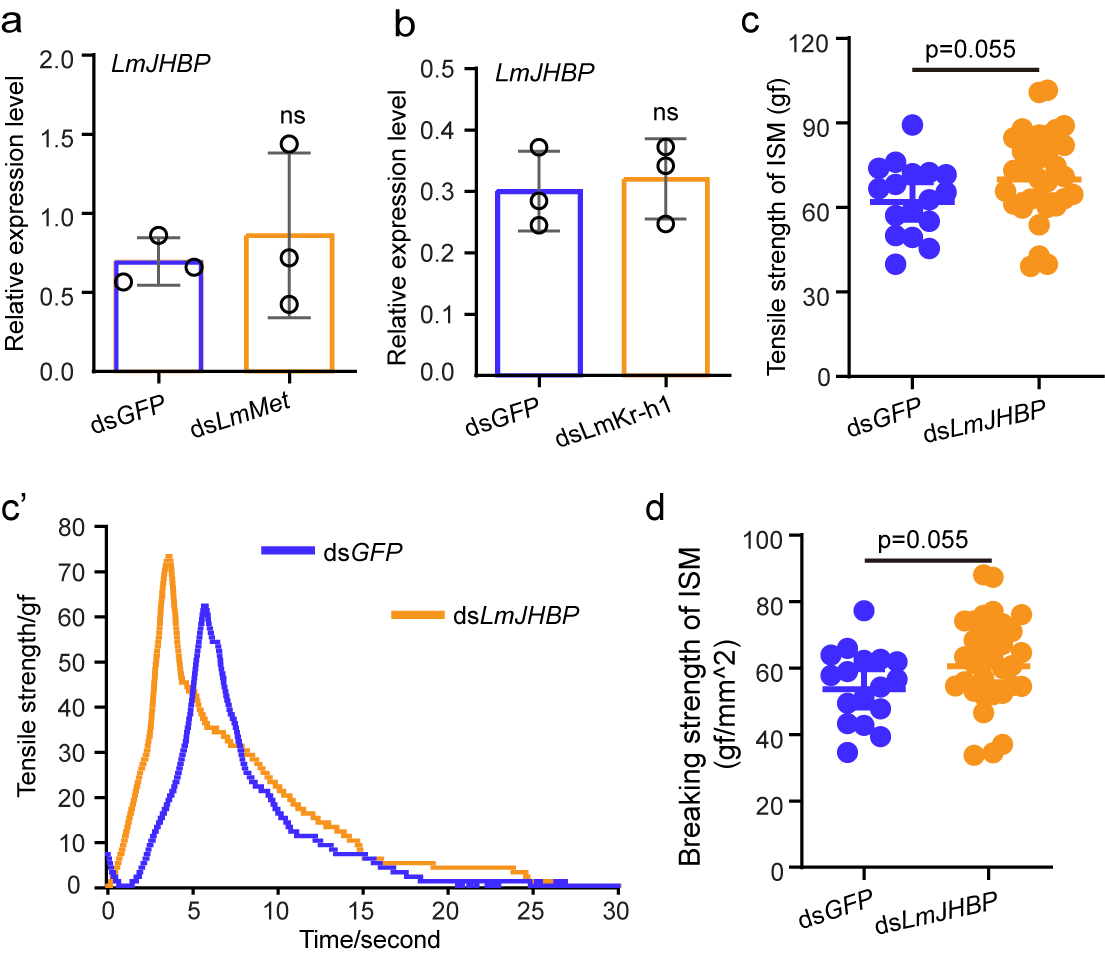

Supplement: S18 Fig — (a, b) The expression level of LmJHBP after injection of dsGFP, dsLmMet, and dsLmKr-h1. (c, c′) Tensile strength of female ISM5 after knockdown of LmJHBP. n ≥ 17 biologically independent locusts. (d) Breaking strength of female ISM5 after knockdown of LmJHBP. n ≥ 17 biologically independent locusts. Student t test (two-tailed) was applied for two-group comparisons. The data are shown as the mean ± SEM. ns, no significant difference. The data underlying the graphs shown in the figure can be found in S2 Data. (TIF) [file pbio.3003321.s018.tif]

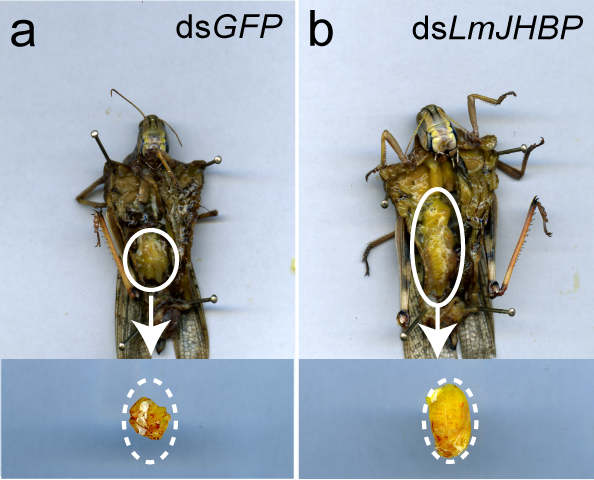

Supplement: S19 Fig — (a, b) The size of the remaining oocysts in adult female locusts after injection of dsLmJHBP compared to that of control. (TIF) [file pbio.3003321.s019.tif]

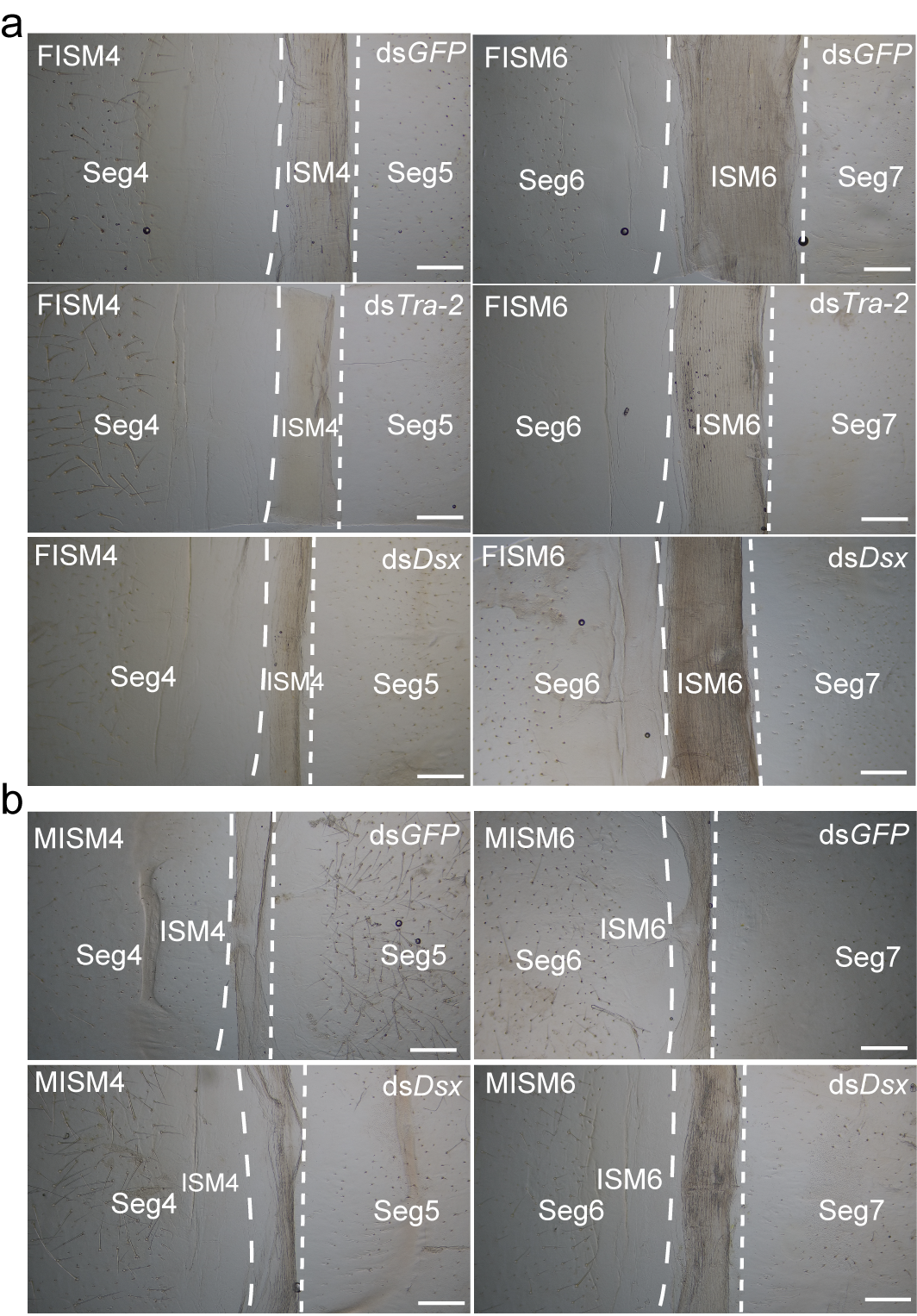

Supplement: S20 Fig — (a) The microstructure difference of female ISM4 and ISM6 between Tra-2 or Dsx RNAi treatment and control groups. (b) The microstructure difference of male ISM4 and ISM6 between Dsx RNAi treatment and control groups. (TIF) [file pbio.3003321.s020.tif]
