# Supplementary material for: The Tra/Dsx-JHBP axis controls female-specific gene expression and oviposition in locusts
Source: PLoS Biol. 2025 Aug 5;23(8):e3003321. doi: 10.1371/journal.pbio.3003321 (PMC12349703; doi:10.1371/journal.pbio.3003321)
Supplement: S1 Table — (DOCX) [file pbio.3003321.s026.docx]

Table S1 Primer sequences used in this study

| Name | Primer sequences (5′-3′) | Application | Length of product (bp) |
| --- | --- | --- | --- |
| ds*GFP* | F: TAATACGACTCACTATAGGGTGGAGAGGGTGAAGG | dsRNA | 571 |
|  | R: TAATACGACTCACTATAGGGGGGCAGATTGTGTGGAC |  |  |
| ds*LmJHBP* | F: TAATACGACTCACTATAGGGCCTCAGCGACGTCATAGTCA |  | 476 |
|  | R:TAATACGACTCACTATAGGGACACGTCAGGGAAGAGGATG |  |  |
| ds*LmAbd-1* | F:TAATACGACTCACTATAGGGCCATCCCGCAGACCTCCAACG |  | 362 |
|  | R:TAATACGACTCACTATAGGGTCAGACCCTCCTGGCCGGGG |  |  |
| ds*LmAbd-6* | F:TAATACGACTCACTATAGGGATGAAGCTGCTGCTCGTTGTC |  | 213 |
|  | R:TAATACGACTCACTATAGGGGCCGCGGACCTCGATGGCCT |  |  |
| ds*Tra-2* | F: TAATACGACTCACTATAGGGACGCTCATACAGTCCAAGGG |  | 462 |
|  | R: TAATACGACTCACTATAGGGCCTTTCTGTCTACCACCCCA |  |  |
| ds*Dsx* | F: TAATACGACTCACTATAGGGCAGCACCCCCTGCCGCTGATG |  | 258 |
|  | R: TAATACGACTCACTATAGGGGATGCGGTTGTACGCTTCTT |  |  |
| ds*LmMet* | F: TAATACGACTCACTATAGGGTTAGGGCAGCATCAGAAAG |  | 421 |
|  | R: TAATACGACTCACTATAGGGTCGTCGGGAGGAAGTGTAT |  |  |
| ds*LmKr-h1* | F: TAATACGACTCACTATAGGGCAGCCGCAGCATCAACAACC |  | 474 |
|  | R:AATACGACTCACTATAGGGCTCGTGAGAAGTCCCAGTGTCC |  |  |
| *β-actin* | F: CGAAGCACAGTCAAAGAGAGGTA | RT-qPCR | 156 |
|  | R: GCTTCAGTCAAGAGAACAGGATG |  |  |
| *LmAbd-1* | F: TTCCCCAAGTACAACCCCATC |  | 138 |
|  | R: GCGAACTGGCACGATGGC |  |  |
| *LmAbd-2* | F: GTGTTTGACCCTGCTGGTG |  | 106 |
|  | R: CCGTTGCCGTTTCGTATT |  |  |
| *LmAbd-6* | F: GACGGCAAGGACTACAGCA |  | 180 |
|  | R: TCGAGTGAGGAGGGAGGAA |  |  |
| *LmAbd-8* | F: AGCAGCCGGGCAACAACC |  | 150 |
|  | R: CGTCACATCAGCAACAGCGAAG |  |  |
| *LmAbd-9* | F: AGGACCTCAGCACCCCAACC |  | 141 |
|  | R: CGGGACAAGCCCAAAAGAGT |  |  |
| *LmGPL* | F: ACAGGTTTGCTGCCTATTT |  | 124 |
|  | R: CCATTGGTTCCAGGTTCTC |  |  |
| *LmGfat* | F: CGTGGAGCCCTAATTGTTGGT |  | 185 |
|  | R: TGAAGAGAGATTCTGTCCTCACTGAG |  |  |
| *LmGNA* | F: CAGCGGGAAGCACATGATCT |  | 122 |
|  | R: TCCAGGGTTTACTGCAGTGACA |  |  |
| *LmAGM* | F: CGCATTGTCTATTTCTTCA |  | 113 |
|  | R: ATCACCACCTGTGCTCCTT |  |  |
| *LmUAP1* | F: TACGGGACCGTAAGGTGTTGG |  | 139 |
|  | R: CCACATTCTGCATTTTTGCTTATAC |  |  |
| *LmCHS1* | F: CTTGAGCCAATTGGTTTGGT |  | 121 |
|  | R: TGAGTTCTGTGGATGCAAGG |  |  |
| *LmFAS3* | F: TCACTGGAACGGAAACGAAA |  | 195 |
|  | R: CCATAGCAAATGCAAAGGGT |  |  |
| *LmELO3* | F: TCTTGACACTGCCAAATGC | RT-qPCR | 101 |
|  | R: TTTCTCCTGAACGGACTGA |  |  |
| *LmDesat2* | F: GCTAGTAGAAGGAAAGAAAT |  | 110 |
|  | R: ATAAACTGAACAAGCACCA |  |  |
| *LmapoLpI/II* | F: AGCGATTTCATCCGGTGGC |  | 191 |
|  | R: GGTGTATGTCTGTCCCTTTT |  |  |
| *LmapoLpIII* | F: ACGCTGCTCGCAGTCCTC |  | 83 |
|  | R: ACCGCCTCCGCGATGTT |  |  |
| *LmLac2* | F: GGTGCTGCTCAGCGATTGG |  | 102 |
|  | R: TCCTGGAACTGGCCCTTGC |  |  |
| *LmAANAT-1* | F: CGGCTTCCAGCAGGTGTT |  | 160 |
|  | R: GCAGTCCTCCACTGATGTTTT |  |  |
| *LmADC* | F: GCTTGTTGTGCCCAGAGT |  | 189 |
|  | R: GCTTGTTGTGCCCAGAGT |  |  |
| *LmEbony* | F: GATTATTCTCGCCTCTACCG |  | 168 |
|  | R: CACAACACCCTTATCCACTCC |  |  |
| *LmTan* | F: GGTCAGGAGGTTCTTGGA |  | 103 |
|  | R: AACGACCTTGTGGTGAAT |  |  |
| *LmJHBP* | F: TCAAGATATGCAGCAGGAACG |  | 130 |
|  | R: GGCACTACGAACGGGTCA |  |  |
| *LmMet* | F: CCACTTACCGGCTTGCTA |  | 144 |
|  | R: GCCCTTCTTCACCTTCTT |  |  |
| *LmKr-h1* | F: CCTACTCGTGCGACATCTGCG |  | 192 |
|  | R: CCTGCTGCTGCTGTTGCTG |  |  |
| *Tra-2* | F: TCTTCCAAGGCATGAGTGA |  | 119 |
|  | R: ACGGCTCCTGGATTGTTCT |  |  |
| *Dsx* | F: TTAATTCATTTCCCTCCACC |  | 119 |
|  | R: CTAGCGTCCGACGAGCCTAT |  |  |
| *LmAbd-1* | F: CATCCCGCAGACCTCCAA | Verify the total length | 926 |
|  | R: GGGCTGCCAAGTCAAGGAG |  |  |
| *LmAbd-6* | F: GTCATCCTGGAGCAGAGCA |  | 624 |
|  | R: TCGTCGAGTGAGGAGGGA |  |  |
| *LmAbd-1* | F: CGCGGATCCATGGCGTTGTGCGGCGCGGCGG | Prokaryotic expression | 552 |
|  | R: CCCAAGCTTTCAGACCCTCCTGGCCGGGG |  |  |
| *LmAbd-6* | F: CGCGGATCCATGATCGCTGTCGCCGCCGCCAG |  | 273 |
|  | R: CCCAAGCTTCTACTGGTTGTACTGGGGCT |  |  |
