# Supplementary material for: The Tra/Dsx-JHBP axis controls female-specific gene expression and oviposition in locusts
Source: PLoS Biol. 2025 Aug 5;23(8):e3003321. doi: 10.1371/journal.pbio.3003321 (PMC12349703; doi:10.1371/journal.pbio.3003321)
Supplement: S2 Table — (DOCX) [file pbio.3003321.s027.docx]

**S2 Table Species and GenBank accession number for phylogenetic tree used in this study**

| Order | | Species | | Protein name | | GenBank accession number | |
| --- | --- | --- | --- | --- | --- | --- | --- |
| Orthoptera | | *Locusta migratoria* | | LmJHBP | | WUG47079.1 | |
|  | | *Schistocerca nitens* | | CnJHBP | | XP_049802128.1 | |
|  | | *Schistocerca cancellata* | | TsJHBP | | XP_049774148.1 | |
|  | | *Schistocerca serialis cubense* | | AsJHBP | | XP_049949963.1 | |
|  | | *Schistocerca gregaria* | | SgJHBP | | XP_049851988.1 | |
|  | | *Schistocerca americana* | | SaJHBP | | XP_046991164.1 | |
|  | | *Schistocerca piceifrons* | | XbJHBP | | XP_047108517.1 | |
| Blattaria | | *Cryptotermes secundus* | | Cstakeout | | XP_023713802.1 | |
|  | | *Reticulitermes flavipes* | | Rfdeviate | | ADM18966.1 | |
|  | | *Zootermopsis nevadensis* | | Zntakeout-like | | XP_021924751.1 | |
|  | | *Pediculus humanus corporis* | | takeout precursor | | XP_002431552.1 | |
| Hemiptera | | *Copidosoma floridanum* | | Cftakeout-like | | XP_023245284.1 | |
|  | | *Bemisia tabaci* | | Bttakeout-like | | XP_018916203.1 | |
|  | | *Sipha flava* | | Sftakeout-like | | *XP_025408311.1* | |
|  | | *Myzus persicae* | | Mptakeout-like | | XP_022176605.1 | |
|  | | *Rhopalosiphum maidis* | | Rmtakeout-like | | XP_026822606.1 | |
|  | | *Aphis craccivora* | | Actakeout-like | | KAF0757685.1 | |
|  | | *Melanaphis sacchari* | | Mstakeout-like | | XP_025202047.1 | |
| Lepidoptera | | *Bombyx mori* | | BmJHBP | | NP_001036947.1 | |
|  | | *Plutella xylostella* | | Pxtakeout | | XP_011561006.1 | |
|  | | *Papilio xuthus* | | Pxtakeout-like | | BAM18288.1 | |
| Orthoptera | | *Locusta migratoria* | | LmAbd-1 | | ASQ42721.1 | |
|  | | *Schistocerca gregaria* | | SgAbd-1 | | XP_049839801.1 | |
|  | | *Schistocerca americana* | | SaAbd-1 | | XP_046980167.1 | |
|  | | *Schistocerca nitens* | | SnAbd-1 | | XP_049794207.1 | |
|  | | *Schistocerca piceifrons* | | SpAbd-1 | | XP_047098085.1 | |
|  | | *Schistocerca cancellata* | | ScAbd-1 | | XP_049767147.1 | |
|  | | *Schistocerca serialis cubense* | | SsAbd-1 | | XP_049941520.1 | |
|  | | *Oxya chinensis* | | OcAbd-1 | | PP833621 | |
|  | | *Acrida cinerea* | | AcAbd-1 | | PP833619 | |
|  | | *Atractomorpha sinensis* | | AsAbd-1 | | PP833620 | |
|  | | *Ceracris nigricornis* | | CnAbd-1 | | GHNZ01020835.1 | |
|  | | *Xenocatantops brachycerus* | | XbAbd-1 | | OFSG01018755.1 | |
|  | | *Tetrix subulata* | | TsAbd-1 | | GASQ02002215.1 | |
|  | | *Atractomorpha sp.* | | AsAbd-1 | | GDYX01022495.1 | |
|  | | *Mecopoda elongata* | | MeAbd-1 | | GDEE01052860.1 | |
| Hymenoptera | | *Camponotus floridanus* | | CfAbd-1 | | XP_011267203.2 | |
|  | | *Lasius niger* | | LnAbd-1 | | KMQ97685.1 | |
|  | | *Formica exsecta* | | FeAbd-1 | | XP_029667540.1 | |
|  | | *Pseudomyrmex gracilis* | | PgAbd-1 | | XP_020289362.1 | |
|  | | *Dufourea novaeangliae* | | DnAbd-1 | | XP_015433792.1 | |
|  | | *Linepithema humile* | | LhAbd-1 | | XP_012233546.1 | |
|  | | *Cephus cinctus* | | CcAbd-1 | | XP_015604921.1 | |
|  | | *Harpegnathos saltator* | | HsAbd-1 | | EFN78517.1 | |
|  | | *Bombus terrestris* | | BtAbd-1 | | XP_003394729.1 | |
|  | | *Melipona quadrifasciata* | | MqAbd-1 | | KOX67424.1 | |
|  | | *Eufriesea mexicana* | | EmAbd-1 | | OAD56753.1 | |
|  | | *Trachymyrmex septentrionalis* | | TsAbd-1 | | XP_018339924.1 | |
|  | *Dinoponera quadriceps* | | DqAbd-1 | | XP_014488065.1 | |  |
|  | *Megachile rotundata* | | MrAbd-1 | | XP_012135502.1 | |  |
|  | *Osmia bicornis bicornis* | | ObbAbd-1 | | XP_029039673.1 | |  |
|  | *Orussus abietinus* | | OaAbd-1 | | XP_012282315.1 | |  |
|  | *Apis cerana cerana* | | AccAbd-1 | | PBC25532.1 | |  |
|  | *Apis mellifera* | | AmAbd-1 | | XP_001120518.2 | |  |
|  | *Apis dorsata* | | AdAbd-1 | | XP_006624959.1 | |  |
|  | *Polistes canadensis* | | PcAbd-1 | | XP_014605526.1 | |  |
|  | *Pogonomyrmex barbatus* | | PbAbd-1 | | XP_011639331.1 | |  |
|  | *Temnothorax longispinosus* | | TlAbd-1 | | TGZ52422.1 | |  |
|  | *Trachymyrmex cornetzi* | | TcAbd-1 | | XP_018363717.1 | |  |
|  | *Atta colombica* | | AcAbd-1 | | XP_018056065.1 | |  |
|  | *Trachymyrmex zeteki* | | TzAbd-1 | | KYQ51945.1 | |  |
|  | *Cyphomyrmex costatus* | | CcAbd-1 | | KYN08652.1 | |  |
|  | *Acromyrmex echinatior* | | AeAbd-1 | | EGI60085.1 | |  |
|  | *Camponotus floridanus* | | CfAbd-1 | | EFN60841.1 | |  |
| Orthoptera | *Locusta migratoria* | | LmAbd-6 | | ASQ42726.1 | |  |
|  | *Schistocerca gregaria* | | SgAbd-6 | | XP_049839301.1 | |  |
|  | *Schistocerca americana* | | SaAbd-6 | | XP_046979765.1 | |  |
|  | *Schistocerca nitens* | | SnAbd-6 | | XP_049794049.1 | |  |
|  | *Schistocerca cancellata* | | ScAbd-6 | | XP_049765448.1 | |  |
|  | *Oxya chinensis* | | OcAbd-6 | | PP833624 | |  |
|  | *Xenocatantops brachycerus* | | XbAbd-6 | | OFSG01016671.1 | |  |
|  | *Tetrix subulata* | | TsAbd-6 | | GASQ02019869.1 | |  |
|  | *Ceracris nigricornis* | | CnAbd-6 | | GHNZ01010936.1 | |  |
|  | *Atractomorpha sp.* | | AsAbd-6 | | GDYX01017872.1 | |  |
|  | *Acrida cinerea* | | AcAbd-6 | | PP833622 | |  |
|  | *Atractomorpha sinensis* | | AsAbd-6 | | PP833623 | |  |
|  | *Gampsocleis gratiosa* | | GgAbd-6 | | GFSE01059448.1 | |  |
|  | *Gryllus bimaculatus* | | GbAbd-6 | | GFMG02074998.1 | |  |
|  | *Mecopoda elongata* | | MeAbd-6 | | GDEE01028814.1 | |  |
| Diptera | *Bactrocera oleae* | | BoAbd-6 | | XP_014094905.1 | |  |
|  | *Drosophila obscura* | | DoAbd-6 | | XP_022218685.1 | |  |
|  | *Ceratitis capitata* | | CcAbd-6 | | XP_004524507.1 | |  |
|  | *Rhagoletis zephyria* | | RzAbd-6 | | XP_017470796.1 | |  |
|  | *Zeugodacus cucurbitae* | | ZcAbd-6 | | XP_011186135.1 | |  |
|  | *Drosophila takahashii* | | DtAbd-6 | | XP_017010529.1 | |  |
|  | *Drosophila eugracilis* | | DeAbd-6 | | XP_017071035.1 | |  |
|  | *Scaptodrosophila lebanonensis* | | SlAbd-6 | | XP_030386120.1 | |  |
|  | *Drosophila persimilis* | | DpAbd-6 | | XP_026847882.1 | |  |
|  | *Drosophila willistoni* | | DwAbd-6 | | XP_002061947.1 | |  |
|  | *Drosophila hydei* | | DhAbd-6 | | XP_023172388.2 | |  |
|  | *Drosophila biarmipes* | | DbAbd-6 | | XP_016956221.1 | |  |
|  | *Drosophila melanogaster* | | Acp65a | | NP_477282.2 | |  |
|  | *Drosophila erecta* | | DeAbd-6 | | XP_001972088.1 | |  |
|  | *Drosophila ficusphila* | | DfAbd-6 | | XP_017048566.1 | |  |
|  | *Stomoxys calcitrans* | | ScAbd-6 | | XP_013110240.1 | |  |
|  | *Lucilia cuprina* | | LcAbd-6 | | XP_023294730.1 | |  |
|  | *Bactrocera dorsalis* | | BdAbd-6 | | XP_011212866.1 | |  |
|  | *Drosophila novamexicana* | | DnAbd-6 | | XP_030560979.1 | |  |
|  | *Drosophila kikkawai* | | DkAbd-6 | | XP_017017864.1 | |  |
|  | *Drosophila rhopaloa* | | DrAbd-6 | | XP_016975764.1 | |  |
|  | *Drosophila elegans* | | DeAbd-6 | | XP_017121847.1 | |  |
|  | *Drosophila arizonae* | | DaAbd-6 | | XP_017862964.1 | |  |
|  | *Drosophila suzukii* | | DsAbd-6 | | XP_016933660.1 | |  |
|  | *Drosophila navojoa* | | DnAbd-6 | | XP_017957013. | |  |
|  | *Drosophila serrata* | | DsAbd-6 | | XP_020808977.1 | |  |
